# Neurological Symptoms and Cause of Death Among Young Children in Low- and Middle-Income Countries

**DOI:** 10.1001/jamanetworkopen.2024.31512

**Published:** 2024-09-03

**Authors:** Sara Ajanovic, Zachary J. Madewell, Shams El Arifeen, Emily S. Gurley, Mohammad Zahid Hossain, Kazi Munisul Islam, Afruna Rahman, Nega Assefa, Lola Madrid, Mohammednur Abdulahi, Kitiezo Aggrey Igunza, Florence Murila, Gunturu Revathi, Mugah Christopher, Samba O. Sow, Karen L. Kotloff, Milagritos D. Tapia, Cheik Bougadari Traor, Inacio Mandomando, Elisio Xerinda, Rosauro Varo, Milton Kincardett, Ikechukwu U. Ogbuanu, Phillip Nwajiobi-Princewill, Alim Swarray-Deen, Ronita Luke, Shabir A. Madhi, Sana Mahtab, Ziyaad Dangor, Jeanie du Toit, Victor Akelo, Portia Mutevedzi, Beth A. Tippett Barr, Dianna M. Blau, Cynthia G. Whitney, Quique Bassat

**Affiliations:** 1 Barcelona Institute for Global Health, Barcelona, Spain; 2 University of Barcelona, Barcelona, Spain; 3 Centro de Investigaçao em Saúde de Manhiça, Manhiça, Mozambique; 4 Global Health Center, US Centers for Disease Control and Prevention, Atlanta, Georgia; 5 International Center for Diarrhoeal Diseases Research, Dhaka, Bangladesh; 6 Department of Epidemiology, The Johns Hopkins Bloomberg School of Public Health, Baltimore, Maryland; 7 College of Health and Medical Sciences, Haramaya University, Harar, Ethiopia; 8 Department of Infectious Disease Epidemiology, London School of Hygiene and Tropical Medicine, London, United Kingdom; 9 Ayder Specialized Comprehensive Hospital, Mekelle University, Mekelle, Ethiopia; 10 Addis Ababa University, Addis Ababa, Ethiopia; 11 Kenya Medical Research Institute, Kisumu, Kenya; 12 Department of Pathology, Aga Khan University Hospital, Nairobi, Kenya; 13 Kisumu County of Department of Health, Kisumu, Kenya; 14 Centre pour le Développement des Vaccins, Ministère de la Santé, Bamako, Mali; 15 Center for Vaccine Development and Global Health, University of Maryland School of Medicine, Baltimore; 16 Instituto Nacional de Saude, Maputo, Mozambique; 17 Crown Agents, Freetown, Sierra Leone; 18 World Hope International, Freetown, Sierra Leone; 19 Department of Medical Microbiology, National Hospital Abuja, Abuja, Nigeria; 20 Department of Obstetrics and Gynaecology, University of Ghana Medical School, Accra, Ghana; 21 Hubert Department of Global Health, Rollins School of Public Health, Emory University, Atlanta, Georgia; 22 South African Medical Research Council Vaccines and Infectious Diseases Analytics Research Unit, Infectious Diseases and Oncology Research Institute, Faculty of Health Sciences, University of the Witwatersrand, Johannesburg, South Africa; 23 Department of Paediatrics and Child Health, Faculty of Health Sciences, University of the Witwatersrand, Johannesburg, South Africa; 24 US Centers for Disease Control and Prevention, Nairobi and Kisumu, Kenya; 25 Nyanja Health Research Institute, Salima, Malawi; 26 Institució Catalana de Recerca i Estudis Avançats, Barcelona, Spain; 27 Pediatrics Department, Hospital Sant Joan de Déu, University of Barcelona, Barcelona, Spain; 28 Centro de Investigación Biomédica en Red de Epidemiología y Salud Pública, Instituto de Salud Carlos III, Madrid, Spain

## Abstract

**Importance:**

The emergence of acute neurological symptoms in children necessitates immediate intervention. Although low- and middle-income countries (LMICs) bear the highest burden of neurological diseases, there is a scarcity of diagnostic and therapeutic resources. Therefore, current understanding of the etiology of neurological emergencies in LMICs relies mainly on clinical diagnoses and verbal autopsies.

**Objective:**

To characterize the association of premortem neurological symptoms and their management with postmortem-confirmed cause of death among children aged younger than 5 years in LMICs and to identify current gaps and improve strategies to enhance child survival.

**Design, Setting, and Participants:**

This cross-sectional study was conducted between December 3, 2016, and July 22, 2022, at the 7 participating sites in the Child Health and Mortality Prevention Surveillance (CHAMPS) network (Bangladesh, Ethiopia, Kenya, Mali, Mozambique, Sierra Leone, and South Africa). Minimally invasive tissue sampling was performed at the CHAMPS sites with specimens from deceased children aged younger than 5 years. This study included deceased children who underwent a premortem neurological evaluation and had a postmortem-confirmed cause of death. Data analysis was performed between July 22, 2022, and January 15, 2023.

**Main Outcomes and Measures:**

Descriptive analysis was performed using neurological evaluations from premortem clinical records and from postmortem determination of cause of death (based on histopathology, microbiological testing, clinical records, and verbal autopsies).

**Results:**

Of the 2127 deaths of children codified during the study period, 1330 (62.5%) had neurological evaluations recorded and were included in this analysis. The 1330 children had a median age of 11 (IQR, 2-324) days; 745 (56.0%) were male and 727 (54.7%) presented with neurological symptoms during illness before death. The most common postmortem-confirmed neurological diagnoses related to death were hypoxic events (308 [23.2%]), meningoencephalitis (135 [10.2%]), and cerebral malaria (68 [5.1%]). There were 12 neonates with overlapping hypoxic events and meningoencephalitis, but there were no patients with overlapping meningoencephalitis and cerebral malaria. Neurological symptoms were similar among diagnoses, and no combination of symptoms was accurate in differentiating them without complementary tools. However, only 25 children (18.5%) with meningitis had a lumbar puncture performed before death. Nearly 90% of deaths (442 of 511 [86.5%]) with neurological diagnoses in the chain of events leading to death were considered preventable.

**Conclusions and Relevance:**

In this cross-sectional study of children aged younger than 5 years, neurological symptoms were frequent before death. However, clinical phenotypes were insufficient to differentiate the most common underlying neurological diagnoses. The low rate of lumbar punctures performed was especially worrying, suggesting a challenge in quality of care of children presenting with neurological symptoms. Improved diagnostic management of neurological emergencies is necessary to ultimately reduce mortality in this vulnerable population.

## Key Points

**Question:**

Are premortem neurological symptoms associated with postmortem-confirmed cause of death among children aged younger than 5 years in low- and middle-income countries (LMICs)?

**Findings:**

In this cross-sectional study of 1330 deceased children, premortem neurological symptoms were present in more than half of the cohort, with overlap among common underlying etiologies (hypoxic events, meningoencephalitis, and cerebral malaria). Lumbar punctures were infrequently done premortem.

**Meaning:**

These findings suggest that neurological symptom management among severely ill children in LMICs was mainly based on clinical evaluation, which is insufficient to differentiate the most common underlying etiologies.

## Teaser

This cross-sectional study examines the association of premortem acute neurological symptoms and their management with cause of death among children aged younger than 5 years in low- to middle-income countries (LMICs).

## Introduction

Neurological symptoms of diseases occurring in childhood are considered medical emergencies warranting immediate intervention.^1^ Appropriate management is highly dependent on using the right diagnostic tools. Neurological symptoms in children are varied and not always etiology specific, rendering exclusive reliance on clinical evaluation insufficient for diagnosis. Therefore, tools such as radiographic imaging and laboratory testing, particularly cerebrospinal fluid (CSF) analysis obtained through lumbar puncture (LP), are indispensable for a correct differential diagnosis.^2^

Acute neurological symptoms, such as seizures, occur more frequently in resource-limited settings, possibly due to the higher burden of associated etiologies (eg, HIV opportunistic infections, malaria, malnutrition, and tuberculosis), poor health-seeking behavior, and restricted access to health services.^3,4,5^ Low- and middle-income countries (LMICs) bear the highest burden of neurological diseases in general^6^ and neurotropic infections in particular, which are the most common cause of neurological emergencies in children.^7^ However, LMICs face a substantial shortage of diagnostic and therapeutic resources to effectively manage these conditions.^8^ Lumbar puncture and CSF analysis, which constitute the standard of care for diagnosing meningitis and differentiating it from other neurological emergencies, are rarely performed in most areas in LMICs.^9,10,11^ The absence of diagnostic testing contributes to suboptimal management of acute neurological syndromes and to underestimation of the true incidence of meningitis. Increasing the number of LPs performed is one objective of the World Health Organization (WHO) 2030 global road map to eradicate meningitis and decrease mortality.^9^ Undoubtedly, underdiagnosis and misdiagnosis often lead to inadequate treatment and preventable adverse outcomes, including death.

Despite a notable reduction in neonatal and child mortality over the past 3 decades, diseases leading to neurological symptoms continue to be a substantial cause of death, particularly in LMICs, with sub-Saharan Africa and Asia being heavily affected. Neonatal mortality accounts for nearly half of all deaths among children aged younger than 5 years, with perinatal factors such as birth asphyxia, sepsis, and meningitis being primary causes.^9,12^ Malaria and meningitis remain among the top 5 causes of death among infants and children.^9,12,13,14,15,16,17,18^

In this study, we investigated associations among clinical phenotypes, premortem management decisions, and confirmed cause of death for deceased children aged younger than 5 years enrolled in the Child Health and Mortality Prevention Surveillance (CHAMPS) network who exhibited neurological impairment before death. Our objectives were to understand the incidence of neurological emergencies and their management in severely ill children before death and to detect gaps and propose measures to enhance survival rates.

## Methods

Ethics committees overseeing investigators at each site and the Emory University Institutional Review Board approved overall and site-specific protocols for this cross-sectional study. Written informed consent was obtained from caregivers. This study followed the Strengthening the Reporting of Observational Studies in Epidemiology (STROBE) reporting guideline.

### Demographic Surveillance System

We analyzed data from all 7 participating study sites in the CHAMPS network^19^ in Bangladesh, Ethiopia, Kenya, Mali, Mozambique, Sierra Leone, and South Africa. The study was conducted between December 3, 2016, and July 22, 2022, and aimed to describe the clinical presentation of children before death and provide cause of death information among deceased neonates (aged 0-27 days), postneonatal infants (aged 28 days to <12 months), and children (aged 12-59 months).

### Specimen and Data Collection

The CHAMPS inclusion criteria for enrollment for minimally invasive tissue sampling were described previously.^19^ A standardized protocol^20^ was followed by all sites. Clinical data were collected using a standardized instrument in RedCap, which included specific closed-response fields as well as space for including an open clinical narrative. Premortem management was performed according to each site’s standards, clinical guidelines, and laboratory capacities (CSF testing capacities are shown in eTable 1 in Supplement 1).

The CHAMPS technicians used a standardized approach^21^ to conduct postmortem minimally invasive tissue sampling using needle biopsies from the brain, lungs, heart, and liver. Additionally, blood and CSF as well as nasopharyngeal and rectal swabs were collected. Macroscopic photographs were captured, and anthropometric measurements were recorded. Local and US Centers for Disease Control and Prevention pathologists thoroughly examined the tissue specimens using routine histopathology techniques, specific stains, and immunohistochemistry. To screen for infectious pathogens, lung tissue, blood, and CSF as well as nasopharyngeal and rectal swabs underwent systematic screening using multiplex TaqMan Array Card (Thermo Fisher Scientific) molecular methods, enabling detection of up to 116 organisms.

Blood and CSF samples were cultured at local laboratories. Specific screening tests were conducted (eg, sickle cell status screening), and polymerase chain reaction assays, antigen testing, and GeneXpert (Cepheid) testing were used to detect HIV, malaria, and tuberculosis, respectively. Verbal autopsies were conducted by locally trained interviewers who engaged with caregivers.^19,21,22,23,24^

### Cause of Death Determination

Once all available data and results for each child were compiled, a multidisciplinary panel of specialists (Determination of Cause of Death [DeCoDe] panel) consisting of clinicians (pediatricians and obstetricians), microbiologists, pathologists, and public health specialists reviewed each case. The DeCoDe panelists established a plausible chain of events leading to the child’s death, coding each step according to the *International Classification of Diseases, Tenth Revision* (*ICD-10*) system in line with WHO recommendations,^25^ considering the underlying, morbid, and immediate causes. If the DeCoDe panelists determined a death to be preventable, a list of measures that could have potentially averted the death was recorded (eTable 2 in Supplement 1). The findings and preventive measures were communicated to the families and communities by a local team for future implementation.

### Statistical Analysis

To be included in this analysis, individuals were required to have available clinical records and a documented neurological evaluation before death. We analyzed all data collected and abstracted specific neurological information obtained through either the standardized clinical questionnaire or the clinical narrative. In our study, neurological symptoms encompassed several signs or symptoms, including seizures at presentation (observed by clinicians or reported by caregivers), seizures witnessed during hospitalization, loss of consciousness, altered mental status (characterized by lethargy, confusion, agitation, and listlessness), Blantyre coma score of 4 or lower, focal neurological presentation, nuchal rigidity, positive Brudzinski sign, positive Kernig sign, and bulging fontanelle (with the latter 4 grouped as meningeal signs). Additionally, fever was considered a concomitant sign. We also examined clinical records for available information on premortem testing (including performance of LP and malaria testing) as well as prescription of antibiotics or antimalarial treatment. All *ICD-10* postmortem diagnoses within the chain of events leading to death were included, and we defined the following groups of diagnoses with potential neurological involvement: perinatal asphyxia, neonatal encephalopathy, meningitis, encephalitis, cerebral malaria, epilepsy, poisoning, and other neurological diagnoses (eTable 3 in Supplement 1).

In this analysis, we evaluated the presence of signs and symptoms stratified by age group and site, and we described the most common overlapping clinical presentations. We evaluated associations between the occurrence of any neurological symptom and the following: age, sex assigned at birth, nutritional status, HIV status, and administration of traditional medicine before death. Additionally, we examined the association between the most prevalent clinical phenotypes in each age group and the most common neurological *ICD-10* diagnoses at any point in the chain of events leading to death. We also explored the association between performance of LP and its result, as well as premortem malaria testing. Data summaries included medians, counts, and percentages, and significant differences among groups were assessed using appropriate χ^2^ tests. *P* < .05 (2-tailed) was considered statistically significant. Euler diagrams were designed using R, version 2021.09.2 build 382 (R Project for Statistical Computing), employing an elliptic representation to minimize standard errors in the visual representations. Figures were edited using Illustrator, version 25.4.8 (Adobe). All analyses were conducted in Excel, version 16.54 (Microsoft Corp) and RStudio, version 4.2.3 (R Project for Statistical Computing). Data analysis was performed between July 22, 2022, and January 15, 2023.

## Results

### Demographics and Clinical Presentation

Of the 2127 deaths of patients aged younger than 5 years codified during the study period, 1330 had data meeting the inclusion criteria (as of July 25, 2022) and were included in this analysis (eFigure in Supplement 1). The cohort included 801 neonates (60.2%), 253 infants (19.0%), and 276 children (20.8%). Their median age was 11 (IQR, 2-324) days; 745 (56.0%) were male and 584 (43.9%) were female. More than half of the cohort (727 [54.7%]) presented with at least 1 neurological sign or symptom before death. The occurrence of neurological symptoms was more frequent among children (172 [62.3%]) and infants (155 [61.3%]) than neonates (400 [49.9%]) (*P* < .001; Table 1). South Africa had the largest number of patients in this analysis (365 [27.4%]), followed by Mozambique (278 [20.9%]), Kenya (231 [17.4%]), Bangladesh (157 [11.8%]), Sierra Leone (154 [11.6%]), Mali (77 [5.8%]), and Ethiopia (68 [5.1%]) (Table 2 and eTable 4 in Supplement 1). Among all patients with neurological impairment, 41 of 727 (5.6%) had HIV and 91 of 727 (12.5%) had received traditional medicine, with no significant differences compared with children without neurological signs.

**Table 1.** Characteristics of Parients in the CHAMPS Cohort With Neurological Involvement Before Death^a^

Characteristic	Type of neurological involvement					
	Any neurological sign or symptom^b^	Seizures at presentation	Seizures during hospitalization	Loss of consciousness	Altered mental status^c^	Meningeal signs^d^
Total	727/1330 (54.7)	206/776 (26.5)	301/893 (33.7)	170/1024 (16.6)	472/1030 (45.8)	30/272 (11.0)
Age, median (IQR), d	11 (2-324)	187 (5-728)	13 (3-286)	99 (1-566)	9 (1-325)	72 (4-575)
Age group						
Neonates	400/801 (49.9)	75/476 (15.8)	174/580 (30.0)	77/612 (12.6)	262/605 (43.3)	12/194 (6.2)
Infants	155/253 (61.3)	50/132 (37.9)	58/147 (39.5)	39/190 (20.5)	99/198 (50.0)	8/39 (20.5)
Children	172/276 (62.3)	81/168 (48.2)	69/166 (41.6)	54/222 (24.3)	111/227 (48.9)	10/39 (25.6)
Sex						
Female	319/584 (54.6)	103/356 (28.9)	140/402 (34.8)	68/449 (15.1)	204/448 (45.5)	11/109 (10.1)
Male	408/745 (54.8)	103/419 (24.6)	161/490 (32.9)	102/574 (17.8)	268/581 (46.1)	19/162 (11.7)
Not determined	0/1	0/1	0/1	0/1	0/1	0/1
HIV status						
Positive	41/71 (57.7)	8/40 (20.0)	10/43 (23.3)	9/63 (14.3)	33/62 (53.2)	1/13 (7.7)
Exposed and uninfected	141/240 (58.8)	34/120 (28.3)	71/172 (41.3)	30/179 (16.8)	84/177 (47.4)	5/53 (9.4)
Uninfected or unknown	545/1019 (53.5)	164/616 (26.6)	220/678 (32.4)	131/782 (16.8)	355/791 (44.9)	24/206 (11.7)
Malnourished	99/162 (61.1)	22/108 (20.4)	29/119 (24.3)	20/143 (14.0)	77/144 (53.5)	8/40 (20.0)
Fever	188/298 (63.1)	82/181 (45.3)	91/186 (48.9)	65/252 (25.8)	123/255 (48.2)	11/56 (19.6)
Traditional medicine	91/161 (56.5)	35/108 (32.4)	30/106 (28.3)	30/131 (22.9)	65/133 (48.9)	6/25 (24.0)
Lumbar puncture performed	76/95 (80.0)	21/35 (60.0)	47/67 (70.1)	13/67 (19.4)	42/67 (62.9)	4/16 (25.0)
Antibiotic treatment	583/1024 (56.9)	165/624 (26.4)	269/709 (37.9)	137/846 (16.2)	375/845 (44.4)	21/225 (9.3)
Malaria testing						
Performed	176/301 (58.5)	68/207 (32.9)	79/222 (35.6)	63/261 (24.1)	125/264 (47.3)	14/73 (19.2)
Positive result	60/95 (63.2)	32/56 (57.1)	31/60 (51.7)	29/83 (34.9)	38/84 (45.2)	3/13 (23.1)
Antimalarial treatment	98/155 (63.2)	50/97 (51.5)	51/101 (50.5)	39/126 (31.0)	53/126 (42.1)	1/17 (5.9)

Abbreviation: CHAMPS, Child Health and Mortality Prevention Surveillance.

^a^ Unless noted otherwise, data are presented as No./total No. (%) of patients. Data are shown for the top 5 neurological signs observed. A complete case analysis approach was used for each sign.

^b^ Includes less frequent symptoms, such as focal neurological deficits, decortication, and decerebration posturing, for which specific data are not represented.

^c^ Includes lethargy, confusion, agitation, and listlessness.

^d^ Includes any of the following: nuchal rigidity, positive Brudzinski sign, positive Kernig sign, or bulging fontanelle.

**Table 2.** Patients in the CHAMPS Cohort With Neurological Involvement Before Death or Neurological *ICD-10* Diagnoses Included in the Chain of Events Leading to Death, According to DeCoDe Panels^a^

Site	No. of patients	Neurological sign, No. (%) of patients with neurological involvement						*ICD-10* diagnosis, No. (%) of patients with neurological involvement			
		Any symptom	Seizures at presentation	Seizures during hospitalization	Loss of consciousness	Altered mental status	Meningeal signs	Perinatal asphyxia	Neonatal encephalopathy	Meningo-encephalitis	Cerebral malaria
All sites											
Total	1330	727 (54.7)	206 (26.5)	301 (33.7)	170 (16.6)	472 (45.8)	30 (11.0)	287 (21.6)	66 (5.0)	135 (10.2)	68 (5.1)
Neonates	801	400 (49.9)	75 (15.8)	174 (30.0)	77 (12.6)	262 (43.3)	12 (6.2)	286 (35.7)	64 (8.0)	91 (11.4)	0
Infants	253	155 (61.3)	50 (37.9)	58 (39.5)	39 (20.5)	99 (50.0)	8 (20.5)	1 (0.4)	2 (0.8)	29 (11.5)	11 (4.3)
Children	276	172 (62.3)	81 (48.2)	69 (41.6)	54 (24.3)	111 (48.9)	10 (25.6)	0	0	15 (5.4)	57 (20.7)
Bangladesh											
Total	157	70 (44.6)	18 (15.4)	24 (22.6)	5 (3.7)	57 (42.9)	0	94 (59.9)	15 (9.6)	3 (1.9)	0
Neonates	154	68 (44.2)	18 (15.5)	23 (22.3)	5 (3.8)	56 (43.1)	0	94 (61.0)	15 (9.7)	3 (1.9)	0
Infants	2	2 (100)	0	1 (50.0)	0	1 (50)	0	0	0	0	0
Children	1	0	0	0	0	0	0	0	0	0	0
Ethiopia											
Total	68	30 (44.1)	2 (3.4)	2 (4.1)	7 (11.9)	27 (47.4)	2 (5.4)	18 (26.5)	2 (2.9)	28 (41.2)	0
Neonates	59	25 (42.4)	1 (1.9)	2 (4.5)	5 (9.6)	22 (44.9)	1 (2.9)	18 (30.5)	2 (3.4)	23 (39.0)	0
Infants	2	0	0	0	0	0	0	0	0	2 (100)	0
Children	7	5 (71.4)	1 (25.0)	0	2 (40.0)	5 (83.3)	1 (33.3)	0	0	3 (42.9)	0
Kenya											
Total	231	139 (60.2)	58 (47.2)	59 (44.7)	43 (30.5)	78 (51)	5 (11.6)	19 (8.2)	2 (0.9)	4 (1.7)	35 (15.2)
Neonates	80	40 (50.0)	14 (31.8)	16 (34.0)	7 (15.9)	20 (41.7)	3 (7.0)	19 (23.8)	2 (2.5)	2 (2.5)	0
Infants	77	53 (68.8)	18 (46.2)	21 (47.7)	20 (38.5)	33 (57.9)	1 (2.3)	0	0	1 (1.3)	8 (10.4)
Children	74	46 (62.2)	26 (65.0)	22 (53.7)	16 (35.6)	25 (52.1)	1 (2.3)	0	0	1 (1.4)	17 (23.0)
Mali											
Total	77	41 (53.2)	10 (16.9)	9 (14.1)	12 (19.0)	33 (53.2)	4 (16.0)	11 (14.3)	6 (7.8)	6 (7.8)	1 (1.3)
Neonates	49	24 (49.0)	3 (8.1)	5 (11.4)	6 (14.6)	18 (46.2)	1 (4.0)	11 (22.4)	6 (12.2)	2 (4.1)	0
Infants	16	10 (62.5)	4 (26.7)	1 (9.1)	4 (30.8)	8 (61.5)	2 (8.0)	0	0	2 (12.5)	0
Children	12	7 (58.3)	3 (42.9)	3 (33.3)	2 (22.2)	7 (70.0)	1 (4.0)	0	0	2 (16.7)	1 (8.3)
Mozambique											
Total	278	164 (59.0)	40 (16.9)	49 (20.9)	58 (23.9)	134 (55.6)	12 (13.3)	64 (23.0)	7 (2.5)	5 (1.8)	15 (5.4)
Neonates	170	92 (54.1)	16 (11.4)	26 (17.8)	35 (24.5)	79 (55.6)	3 (3.3)	64 (37.6)	7 (4.1)	3 (1.8)	0
Infants	37	23 (62.2)	5 (16.1)	5 (17.9)	4 (12.5)	19 (57.6)	2 (2.2)	0	0	1 (2.7)	1 (2.7)
Children	71	49 (69.0)	19 (29.2)	18 (30.0)	19 (27.9)	36 (54.5)	7 (7.8)	0	0	1 (1.4)	14 (19.7)
Sierra Leone											
Total	154	74 (48.1)	39 (43.3)	38 (42.7)	23 (18.0)	34 (27.0)	2 (40.0)	30 (19.5)	1 (0.6)	13 (8.4)	27 (17.5)
Neonates	52	22 (42.3)	8 (21.6)	16 (41.0)	3 (7.9)	7 (19.4)	1 (20.0)	30 (57.7)	1 (1.9)	8 (15.4)	0
Infants	36	16 (44.4)	9 (50.0)	7 (36.8)	8 (25.0)	10 (31.2)	1 (20.0)	0	0	4 (11.1)	2 (5.6)
Children	66	36 (54.5)	22 (62.9)	15 (48.4)	12 (20.7)	17 (29.3)	0	0	0	1 (1.5)	25 (37.9)
South Africa											
Total	365	209 (57.3)	39 (41.9)	120 (54.8)	22 (8.6)	109 (42.2)	5 (8.2)	51 (14.0)	33 (9.0)	76 (20.8)	0
Neonates	237	129 (54.4)	15 (30.0)	86 (54.8)	16 (9.9)	60 (37.3)	3 (4.9)	49 (20.7)	31 (13.1)	48 (20.3)	0
Infants	83	51 (61.4)	14 (51.9)	23 (56.1)	3 (5.2)	28 (47.5)	2 (3.3)	2 (2.4)	2 (2.4)	21 (25.3)	0
Children	45	29 (64.4)	10 (62.5)	11 (52.4)	3 (8.3)	21 (55.3)	0	0	0	7 (15.6)	0

Abbreviations: CHAMPS, Child Health and Mortality Prevention Surveillance; DeCoDe, Determination of Cause of Death; *ICD-10*, *International Classification of Diseases, Tenth Revision*.

^a^ A complete case analysis approach was used for each sign and diagnosis.

Altered mental status was reported in 472 of 1030 patients (45.8%). Seizures were present in 206 of 776 patients (26.5%) at presentation and 301 of 893 (33.7%) at hospitalization. Loss of consciousness was present in 170 of 1024 patients (16.6%), with those aged 12 to 59 months (54 of 222 [24.3%]) experiencing it twice as often as neonates (77 [12.6%]) (*P* < .001). Meningeal signs were recorded infrequently in the neurological evaluation (positive for 30 of 272 children [11.0%]).

### Causes of Death and Premortem Management

**Figure 1.**
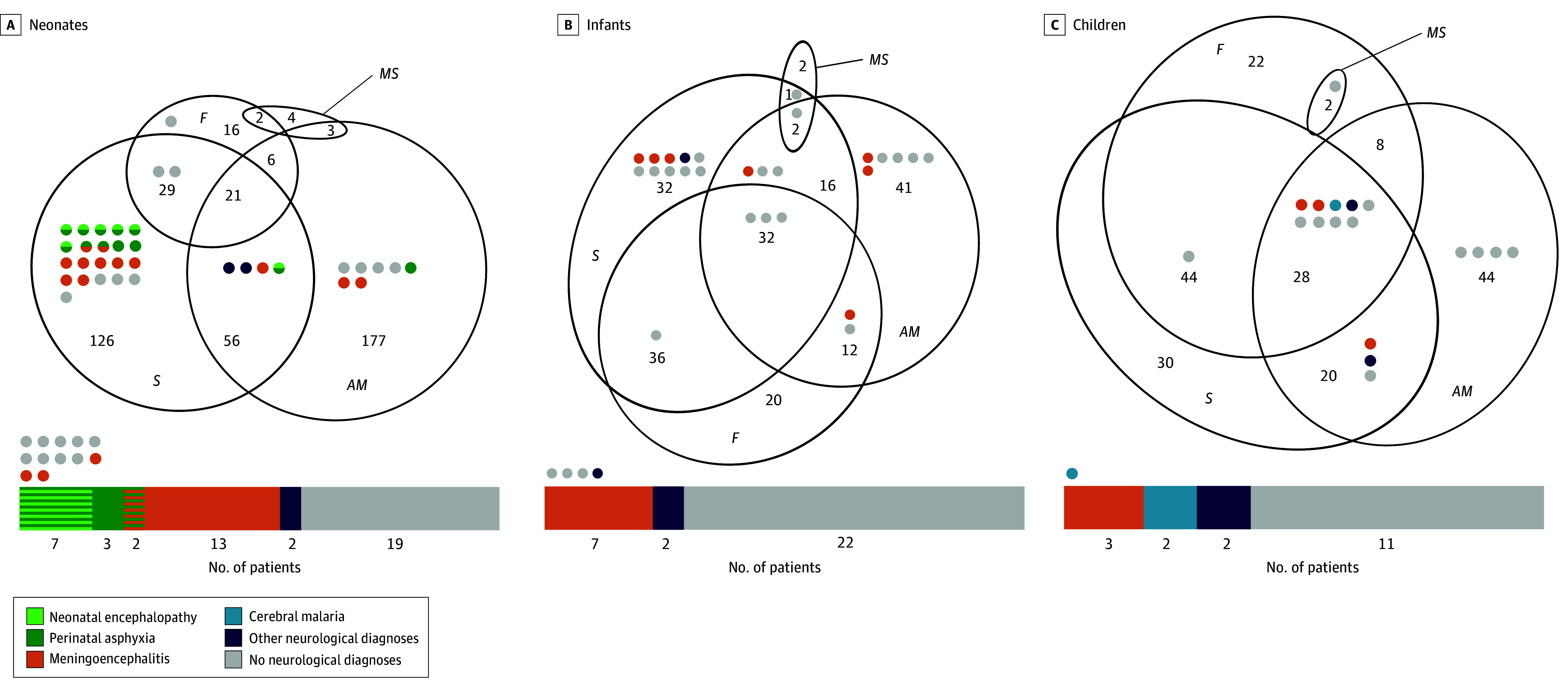
Patients With Neurological Signs and Fever and Their Overlap Euler diagrams are presented for neonates (A), infants (B), and children (C). Patients who went under lumbar puncture (LP) are pinpointed (each point represents 1 LP) according to their clinical presentation. Point colors represent the ultimate neurological diagnosis after evaluation by the DeCoDe panel. The bar beneath each age group represents the proportion of final diagnoses among children who underwent an LP (46 neonates, 31 infants, and 18 children). Dots outside of the bars represent children without any neurological symptoms who underwent an LP. Euler diagram errors: 0.001 in all age groups; stress: less than 0.01 in all age groups. AM indicates altered mental status; DeCoDe, Determination of Cause of Death; F, fever; MS, meningeal sign; and S, seizure.

Neurological signs exhibited overlapping patterns across all age groups (Figure 1). We assessed the accuracy of previously described case definitions for suspected meningitis, cerebral malaria, and neonatal hypoxia^26,27,28^ (eTable 5 in Supplement 1) as well as the combinations of different signs and symptoms associated with them (eTable 6 in Supplement 1).

The presence of neurological signs was more frequent among participants with reported fever (188 of 298 [63.1%]; *P* < .001). Having neurological symptoms was associated with the decision to perform an LP (76 of 95 [80.0%]; *P* < .001), to prescribe antibiotics (583 of 1024 [56.9%]; *P* = .003), and to prescribe antimalarial treatment (98 of 155 [63.2%]; *P* = .03). Overall, LPs were performed before death in 95 of 1330 patients (7.1%), in 77 of 727 (10.6%) with recorded neurological symptoms before death, and in 25 of 135 (18.5%) with postmortem-confirmed meningitis. The capacity to analyze CSF varied across sites (eTable 1 in Supplement 1). Most LPs (83 of 95 [87.4%]) were done in South Africa, with the remainder in Kenya (5 [5.3%]), Mozambique (4 [4.2%]), and Mali (3 [3.2%]). Among neonates and infants, LPs were primarily performed in patients presenting with seizures, altered mental state, or both; in older children, the distribution of signs was more diverse (Figure 1). A total of 423 neonates met criteria for an LP and 89 had postmortem-confirmed meningitis, but only 34 LPs were conducted. The necessary number of tests to detect 1 case of meningitis in neonates was 7.98. Among infants and children, 321 met criteria for an LP, while only 43 LPs were performed; the necessary number of tests was 8.92. Results from these premortem LPs were available for 49 patients; of these, 6 confirmed acute bacterial meningitis, which was also confirmed in postmortem investigation (eTable 7 in Supplement 1).

**Figure 2.**
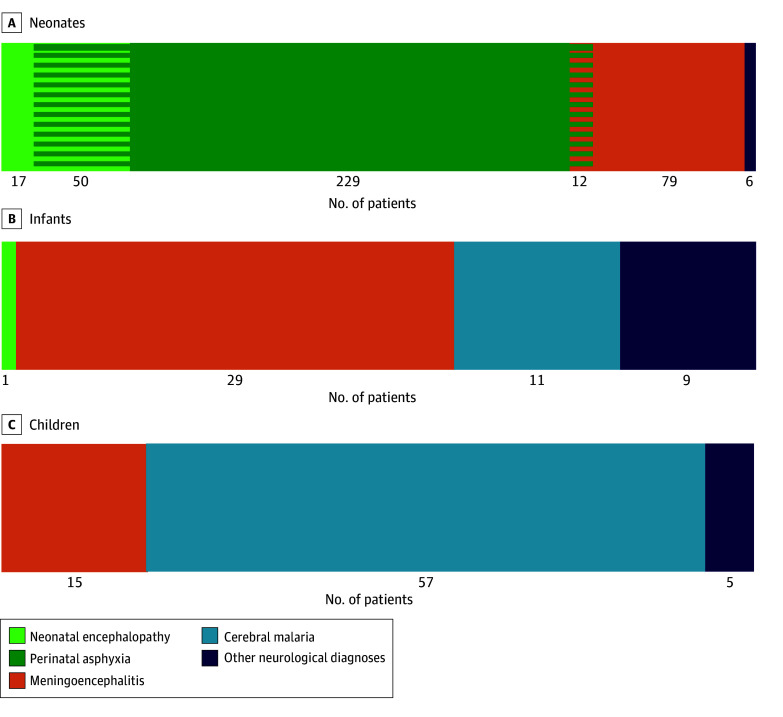
Cause of Death Involving the Central Nervous System, Stratified by Age Group Cause of death is presented for neonates (A), infants (B), and children (C). Data are presented for 520 of 1330 patients (representing 39.1% of the cohort).

In the postmortem *ICD-10* diagnoses determined by the expert panel (eTables 8 and 9 in Supplement 1), more than one-third of children (520 of 1330 [39.1%]) had at least 1 diagnosis affecting the central nervous system. A total of 184 of 520 children (35.4%) did not have documented neurological signs or symptoms. Among the 801 neonates, the most common neurological diagnoses were hypoxic events, including perinatal asphyxia and neonatal encephalopaty (308 [38.5%]), and meningoencephalitis (91 [11.4%]). Among the 253 infants, cerebral malaria was present in 11 (4.3%) and meningoencephalitis was present in 29 (11.5%). Among the 276 children, cerebral malaria was confirmed in 57 (20.7%) and meningoencephalitis was confirmed in 15 (5.4%). There were 12 neonates with overlapping hypoxic events and meningoencephalitis, but there were no patients with overlapping cerebral malaria and meningoencephalitis (Figure 2). Diagnoses of neonatal encephalopathy, meningoencephalitis, and cerebral malaria were associated with the presence of neurological signs and symptoms (eTable 10 in Supplement 1). Patients with noncerebral malaria and cerebral malaria presented with similar neurological symptoms; a high number of seizures was reported in patients with malaria (60 [50.8%]), including 24 (48.0%) with noncerebral malaria and 36 (52.9%) with cerebral malaria (*P* = .73; eTable 11 in Supplement 1). Loss of consciousness was the only symptom that was significantly higher among those with postmortem-confirmed cerebral malaria (eTable 11 in Supplement 1).

Overall, 1 in 5 deaths among children aged 12 to 59 months were attributed to cerebral malaria. Sierra Leone had the highest proportion (25 of 66 [37.9%]), followed by Kenya (17 of 74 [23.0%]), Mozambique (14 of 71 [19.7%]), and Mali (1 of 12 [8.3%]), with the remaining sites reporting no patients with cerebral malaria (Table 2). Malaria testing was performed in 285 patients, and it guided the use of antimalarial treatment. Antibiotics were similarly prescribed in those with both positive and negative malaria test results (eTable 12 in Supplement 1).

### Preventability of Deaths After Neurological Symptoms

The DeCoDe panelists assessed the preventability of deaths on a case-by-case basis. They assessed measures that could have changed the chain of events leading to death, starting with health education, followed by health-seeking behavior and access to health care, the clinical management at presentation, and the quality of care during hospitalization until death (eTable 2 in Supplement 1).

**Figure 3.**
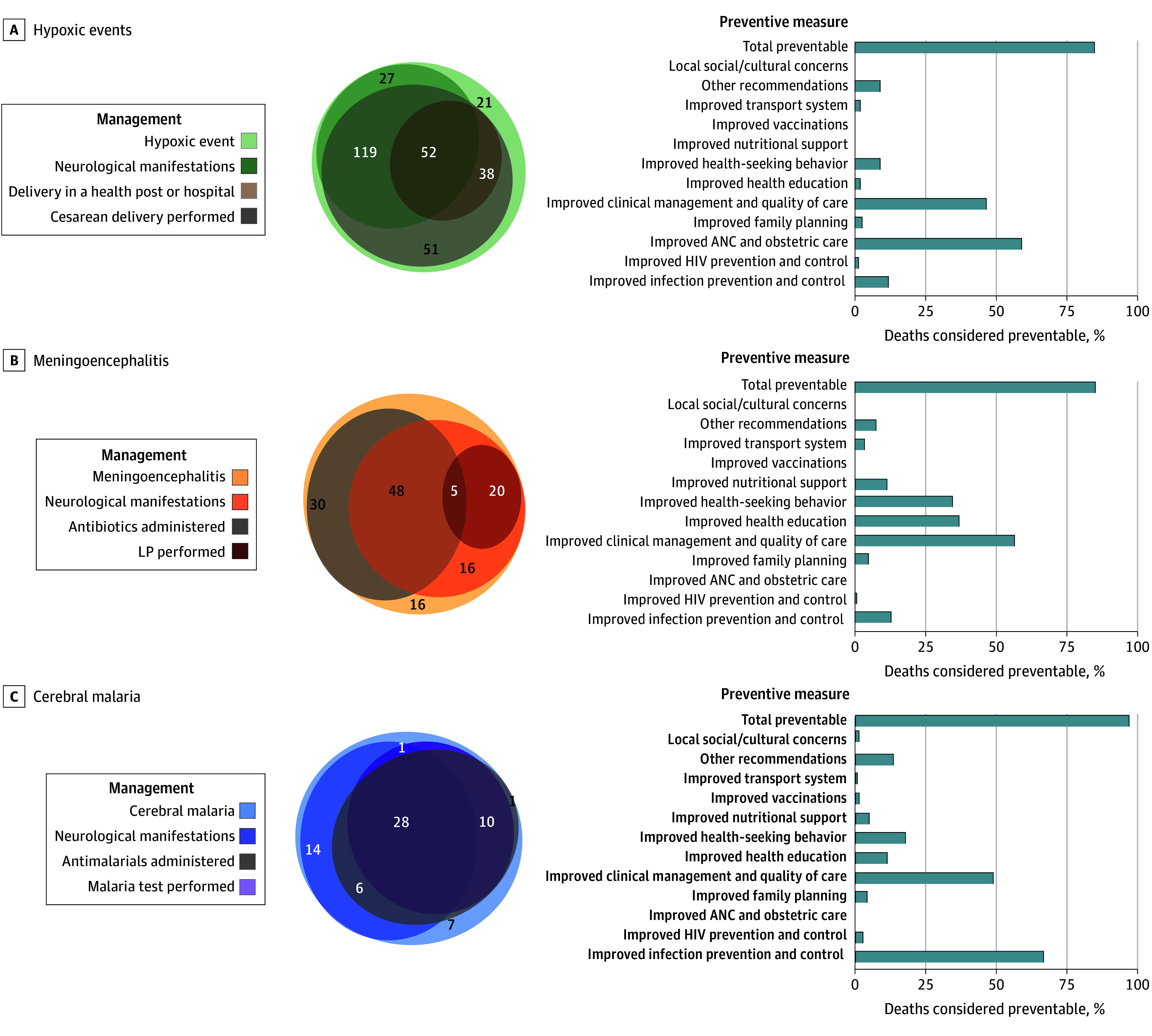
Premortem Management of the 3 Most Common Neurological Diagnoses Present in the Chain of Events Leading to Death and the Proportion of Deaths Considered Preventable Management (left) and preventive measures (right) are presented for patients with hypoxic events (A), meningoencephalitis (B), and cerebral malaria (C). The median number of preventive measures to be implemented in each case of death was 2 (IQR, 1-3) in all 3 groups. ANC indicates antenatal care; LP, lumbar puncture.

Among the 308 neonates with hypoxic events (present in 22.8% of all deaths in the cohort), 243 (80.2%) were delivered in a health post or hospital; 90 (29.7%) were delivered via cesarian section. The DeCoDe panelists considered 257 (83.4%) of these hypoxia-related deaths as preventable, with the primary prevention measure being the improvement of antenatal and obstetric care (eg, to detect fetal stress and act accordingly), followed by improvement in clinical management and quality of care for newborns experiencing the consequences of hypoxic events. Of the meningoencephalitis-related deaths (135 [10.2%]), only 25 patients with postmortem-confirmed meningitis (25 of 135 [18.5%]) had an LP performed before death and 85 (63.0%) received antibiotic treatment. Ethiopia had the lowest number of participants enrolled at the time of the analysis but had the highest proportion of deaths attributed to meningitis (28 of 68 [41.2%]; Table 2), and no LPs were performed before death. The DeCoDe panelists considered nearly all of these meningoencephalitis-related deaths (114 [85.1%]) to be preventable, with improved clinical management and quality of care deemed the most necessary prevention measures (eg, to diagnose meningitis and treat accordingly), followed by improved health education and health-seeking behavior to detect danger signs and seek medical attention. Regarding deaths attributed to cerebral malaria (68 [5.1%]), malaria testing was performed in 42 patients (61.8%) and antimalarial treatment was administered to 48 patients (70.6%). The DeCoDe panelists deemed up to 97.0% of these malaria-related deaths to be preventable, with improvement in clinical management and quality of care as the most important measure required, followed by improving the health education and health-seeking behavior of parents (Figure 3).

## Discussion

The CHAMPS network provides the opportunity to increase knowledge about the underlying etiology of neurological emergencies among children in LMICs. The current lack of understanding is primarily due to the limited availability of diagnostic tools in LMICs to conduct comprehensive etiological studies in living patients with neurological symptoms. The findings of this study suggest that neurological symptoms were common before death among children in the CHAMPS cohort, clinical phenotypes were not enough to differentiate the underlying etiology of neurological symptoms, and necessary testing of CSF was very rarely performed.

### Clinical Symptoms and Causes of Death

More than half of deceased neonates and children in our cohort had evidence of at least 1 neurological sign or symptom before death, which underscores their importance as common symptoms in the pathway to death. Overall, neurological signs were more frequently observed in older children, whereas neurological causes of death were notably more prevalent among neonates, possibly due to the difficulty of recognizing the less specific symptoms of neurological disorders in the latter. Altered mental status was present in nearly half of our cohort, which is twice as frequent as in a previous study involving febrile patients with HIV, whereby its occurrence was associated with a 4-fold increase in the risk of mortality.^29^ Although seizures are known to be common in children from LMICs,^4,30,31,32^ we observed a much higher incidence in this study conducted in a cohort with fatal outcomes. However, we were unable to differentiate severe malaria with febrile seizures from cerebral malaria.^33,34,35,36^ Loss of consciousness was reported in approximately 1 in every 7 patients and has also been recognized as a poor prognostic factor in critically ill children^37^; therefore, it appeared as the best sign to differentiate cerebral malaria from noncerebral malaria. Meningeal signs were evaluated and documented in only a fifth of our cohort, highlighting the need for improvement in the clinical evaluation and recording practices in our specific settings.^38^

Patients exhibiting neurological symptoms demonstrated a higher likelihood of presenting with fever, undergoing an LP, and receiving antimicrobial treatment. This observation supports the recognition by clinicians that infections involving the central nervous system are the leading cause of neurological symptoms in LMICs.^7^ The clinical phenotypes exhibited overlapping characteristics across age groups and final postmortem *ICD-10* diagnoses. Our analysis supports the notion that specific clinical presentations alone cannot reliably establish a definitive diagnosis. This finding is consistent with previous studies conducted in pediatric patients who were alive but had comparatively limited diagnostic information available.^39^

### Neurological Causes of Death, Their Management Before Death, and Preventability

Overall, the most prevalent *ICD-10* neurological diagnoses in our study were hypoxic neonatal events, meningoencephalitis, and cerebral malaria, all of which are among the leading causes of mortality among children aged younger than 5 years globally.^9,12,13,14,15,16,17,18^ Meningoencephalitis emerged as the most common differential diagnosis across all age groups, needing to be differentiated from hypoxia in neonates and from cerebral malaria in infants and children. There were 12 deceased children in this study who had *ICD-10* diagnoses of both a hypoxic event and meningitis, whereas there were no overlapping cases of cerebral malaria and meningitis in the entire cohort. These findings are encouraging to further investigate the overlap between malaria and meningitis in the CHAMPS network because it provides the opportunity for in-depth investigations of both diseases, including CSF and central nervous system pathology testing.

In this study, the leading cause of death in the neonatal period was neonatal hypoxia. A notable number of these deaths occurred despite the mothers giving birth in health facilities. Cesarean delivery, which can reduce the consequences of hypoxic events, was infrequently performed. These deaths were largely considered preventable with enhancements in perinatal clinical management and the quality of care provided.

Regarding meningitis deaths, premortem LPs were performed in less than one-tenth of patients who met the criteria. It is noteworthy that most LPs were conducted in South Africa. Consequently, in 6 of 7 settings, children who died from meningitis were rarely tested before death, indicating that management heavily relied on clinical suspicion and empirical treatment. In fact, Ethiopia, the site with the lowest number of participants enrolled at the time of the analysis, also had the highest proportion of deaths attributed to meningitis (28 of 68 [41.2%]), but no LPs were performed before death. These findings are likely attributable to multiple factors, including the following: lack of resources to perform an LP^5,7,9,30,40^; lack of capacity to test CSF in most of the hospitals from our sites; hesitancy of practitioners and families toward the procedure, especially among patients with very severe illness^10,41^; and lack of clear guidelines including LPs in the routine diagnostic pathway.^10,11^ This problem is particularly concerning among neonates: only 1 in 7 neonates presenting with seizures had an LP performed despite clear WHO guidelines recommending its use.^42^ Furthermore, an LP was performed in only 1 neonate with fever without neurological signs; tools commonly used in high-income countries to rule out potentially severe infections among neonates (eg, blood testing for C-reactive protein or procalcitonin) and therefore avoid LPs are rarely available in LMICs.^43,44^ Neonates were the age group in which most neurological diagnoses were confirmed postmortem, while they were the less symptomatic. Given the extremely complicated task of making a differential diagnosis among neonates based solely on unspecific clinical presentations, it is particularly worrying that the laboratory-based examinations needed to provide a correct diagnosis were seldom available and performed. Despite efforts to increase the performance of an LP in LICs,^9^ the CHAMPS data revealed extremely low compliance with LP implementation in clinical practice in these settings.

Malaria testing before death was not performed for more than one-third of patients with neurological symptoms attributed to cerebral malaria, and antimalarial treatment was not administered to a similar proportion of patients. These findings highlight the need to improve clinical care and management practices as reported by specialist panels, along with the importance of enhancing malaria prevention and control strategies.^45^

### Limitations

This study has some limitations. Our cohort consisted of children who died, and we lacked a control group with better outcomes and comparable comprehensive etiological information for comparison. Also, differences in sites’ recruitment status and in resources and standards of care make comparisons between countries inaccurate, allowing us only to describe them. Another limitation is that clinical records were unavailable for one-third of our cohort, and most community deaths were not included for this reason. This limitation may have biased our results because the most rapidly progressed deaths, which may be more symptomatic, can be missed. Additionally, a considerable proportion of children with definitive neurological *ICD-10* diagnoses did not have any documented positive neurological signs, which is highly improbable and likely reflects inadequate clinical documentation in the hospitals (Garcia Gomez et al, results under review).^38^ The combination of these factors suggests that our results may underestimate the actual incidence of neurological symptoms in severely ill children.

## Conclusions

In this cross-sectional study of severely ill children in LMICs, neurological symptoms were common before death and were associated with potentially treatable diseases. Clinical symptoms of neurological syndromes, even in the most severe cases, overlapped across the most common etiologies (hypoxic events, meningoencephalitis, and cerebral malaria), limiting their discriminatory value. Despite the essential role of an LP in the differential diagnosis, its utilization in our clinical settings was worryingly infrequent. These findings suggest that urgent attention is required to improve early diagnostic tools that enable accurate identification of neurological emergencies in this vulnerable population to ultimately reduce mortality rates among children aged younger than 5 years.

## Notes

Supplement 1. **eTable 1.** Available Resources for Cerebrospinal Fluid (CSF) Testing at Each Study Site **eTable 2.** List of Measures to Prevent Each Death **eTable 3.** Neurological *ICD-10* Diagnoses Included in the Analysis **eFigure.** Flowchart of Participants Included in the Analysis and Reasons for Exclusion **eTable 4.** Participants Enrolled per Year and Country **eTable 5.** Sensitivity and Specificity of Case Definitions for Suspected Meningitis, Cerebral Malaria, and Neonatal Hypoxic Events (Including Both Perinatal Asphyxia and Neonatal Encephalopathy) **eTable 6.** Sensitivity and Specificity of Each Sign and Combination of Signs Associated With the Most Common Neurological Diagnoses **eTable 7.** Characteristics of Participants Who Had a Lumbar Puncture Performed Prior to Death (n = 95) **eTable 8.** Presence of Neurological Symptoms Across All Causes of Death Included in the Chain of Events Leading to Death **eTable 9.** Presence of Neurological Symptoms Across Causes of Death, Grouped by Syndrome **eTable 10.** Signs and Management Decisions Described by Final Neurological Diagnosis (After DeCoDe Panel Discussions) **eTable 11.** Neurological Signs Present in Children With Cerebral Malaria and No-Cerebral Malaria Listed in the Chain of Events Leading to Death **eTable 12.** Malaria Testing in the Cohort, and Prescription of Antimalarial and Antimicrobial Treatment

Supplement 2.
Nonauthor Collaborators

Supplement 3.
Data Sharing Statement
