# Supplementary material for: Neurological Symptoms and Cause of Death Among Young Children in Low- and Middle-Income Countries
Source: JAMA Netw Open. 2024 Sep 3;7(9):e2431512. doi: 10.1001/jamanetworkopen.2024.31512 (PMC11372484; doi:10.1001/jamanetworkopen.2024.31512)
Supplement: Supplement 1. — eTable 1. Available Resources for Cerebrospinal Fluid (CSF) Testing at Each Study Site eTable 2. List of Measures to Prevent Each Death eTable 3. Neurological ICD-10 Diagnoses Included in the Analysis eFigure. Flowchart of Participants Included in the Analysis and Reasons for Exclusion eTable 4. Participants Enrolled per Year and Country eTable 5. Sensitivity and Specificity of Case Definitions for Suspected Meningitis, Cerebral Malaria, and Neonatal Hypoxic Events (Including Both Perinatal Asphyxia and Neonatal Encephalopathy) eTable 6. Sensitivity and Specificity of Each Sign and Combination of Signs Associated With the Most Common Neurological Diagnoses eTable 7. Characteristics of Participants Who Had a Lumbar Puncture Performed Prior to Death (n = 95) eTable 8. Presence of Neurological Symptoms Across All Causes of Death Included in the Chain of Events Leading to Death eTable 9. Presence of Neurological Symptoms Across Causes of Death, Grouped by Syndrome eTable 10. Signs and Management Decisions Described by Final Neurological Diagnosis (After DeCoDe Panel Discussions) eTable 11. Neurological Signs Present in Children With Cerebral Malaria and No-Cerebral Malaria Listed in the Chain of Events Leading to Death eTable 12. Malaria Testing in the Cohort, and Prescription of Antimalarial and Antimicrobial Treatment [file jamanetwopen-e2431512-s001.pdf]

Supplementary Online Content

Ajanovic S, Madewell ZJ, El Arifeen S, et al; the Child Health and Mortality Prevention Surveillance (CHAMPS) Consortium. Neurological symptoms and cause of death among young children in low- and middle-income countries. *JAMA Netw Open*. 2024;7(9):e2431512. doi:10.1001/jamanetworkopen.2024.31512

eTable 1. Available Resources for Cerebrospinal Fluid (CSF) Testing at Each Study Site

eTable 2. List of Measures to Prevent Each Death

eTable 3. Neurological *ICD-10* Diagnoses Included in the Analysis

eFigure. Flowchart of Participants Included in the Analysis and Reasons for Exclusion

eTable 4. Participants Enrolled per Year and Country

eTable 5. Sensitivity and Specificity of Case Definitions for Suspected Meningitis, Cerebral Malaria, and Neonatal Hypoxic Events (Including Both Perinatal Asphyxia and Neonatal Encephalopathy)

eTable 6. Sensitivity and Specificity of Each Sign and Combination of Signs Associated With the Most Common Neurological Diagnoses

eTable 7. Characteristics of Participants Who Had a Lumbar Puncture Performed Prior to Death (n = 95)

eTable 8. Presence of Neurological Symptoms Across All Causes of Death Included in the Chain of Events Leading to Death

eTable 9. Presence of Neurological Symptoms Across Causes of Death, Grouped by Syndrome

eTable 10. Signs and Management Decisions Described by Final Neurological Diagnosis (After DeCoDe Panel Discussions)

eTable 11. Neurological Signs Present in Children With Cerebral Malaria and No-Cerebral Malaria Listed in the Chain of Events Leading to Death

eTable 12. Malaria Testing in the Cohort, and Prescription of Antimalarial and Antimicrobial Treatment

This supplementary material has been provided by the authors to give readers additional information about their work.

© 2024 Ajanovic S et al. *JAMA Network Open.*

eTable 1. Available Resources for Cerebrospinal Fluid (CSF) Testing at Each Study Site

Site	CSF culture	CSF leukocyte count	CSF biochemistry	CSF agglutination tests	CSF molecular testing
Bangladesh		x	x		
Ethiopia	x				
Kenya					
Mali	x*	x*		x*	x**
Mozambique	x		x	x	
Sierra Leone					
South Africa	x	x	x	if indicated	
* Tests available depending on the facility and caregivers' financial resources.					
** Tests available at national level, but unlikely to be incorporated in medical records					

eTable 2. List of Measures to Prevent Each Death

Measures to prevent this death	Improved infection prevention and control
	Improved HIV prevention and control
	Improved antenatal care and obstetric care and management
	Improved family planning
	Improved clinical management and quality of care
	Improved Health education (immunizations, preventing malnutrition, diarrhea, burns, poisoning, etc
	Improved health-seeking behavior
	Improved nutritional support
	Improved vaccination
	Improved transport system
	Other recommendations
	(Open answer): provide with details of how the death could have been prevented to inform families and community about specific determinants to prevent deaths in the future.

A list of potential measures needs to be fulfilled if the panel consideres a death as preventable, and an open answer text must be fulfilled in order to give feedback to families and community regarding how to prevent similar deaths in the future.

© 2024 Ajanovic S et al. *JAMA Network Open.*

eTable 3. Neurological *ICD-10* Diagnoses Included in the Analysis

**ICD-10 neurological diagnoses**	
**Perinatal hypoxic events:**	
**P20**	Intrauterine hypoxia
**P21**	Birth asphyxia
**P91**	Hypoxic encephalopathy
**Meningoencephalitis**	
**G00-G09**	Inflammatory diseases of the central nervous system
**A17**	Tuberculosis of nervous system
**A39·0**	Meningococcal meningitis
**A39·8**	Meningococcal encephalitis
**A39·1**	Meningococcal encephalitis
**A80-A89**	Viral infections of the central nervous system
**A32·1**	Listerial meningiis and meningoencephalitis
**B02·0**	Zoster encephalitis
**B02·1**	Zoster meningitis
**B26·0**	Mumps encephalitis
**B26·1**	Mumps meningitis
**B37·1**	Candidal meningitis
**B45·1**	Criptococcal meningitis
**B01·0**	Varicella meningitis
**B01·1**	Varicella encephalitis
**B06·0**	Rubella meningitis
**B00·3**	Herpesviral meningitis
**B00·4**	Herpesviral encephalitis
**B05·0**	Measles encephalitis
**B05·1**	Measles meningitis
**Cerebral malaria**	
**B50·0**	Plasmodium Falciparum cerebral malaria
**Epilepsy**	
**G40**	Epilepsy
**G41**	Status epilepticus
**Poisoning**	
**T**	Poisoning
**Other neurological diseases**	
**G10-G37**	
**G42-G99**	

The clinical definitions can be found at https://icd.who.int/browse10/2016

© 2024 Ajanovic S et al. *JAMA Network Open.*

eFigure. Flowchart of Participants Included in the Analysis and Reasons for Exclusion

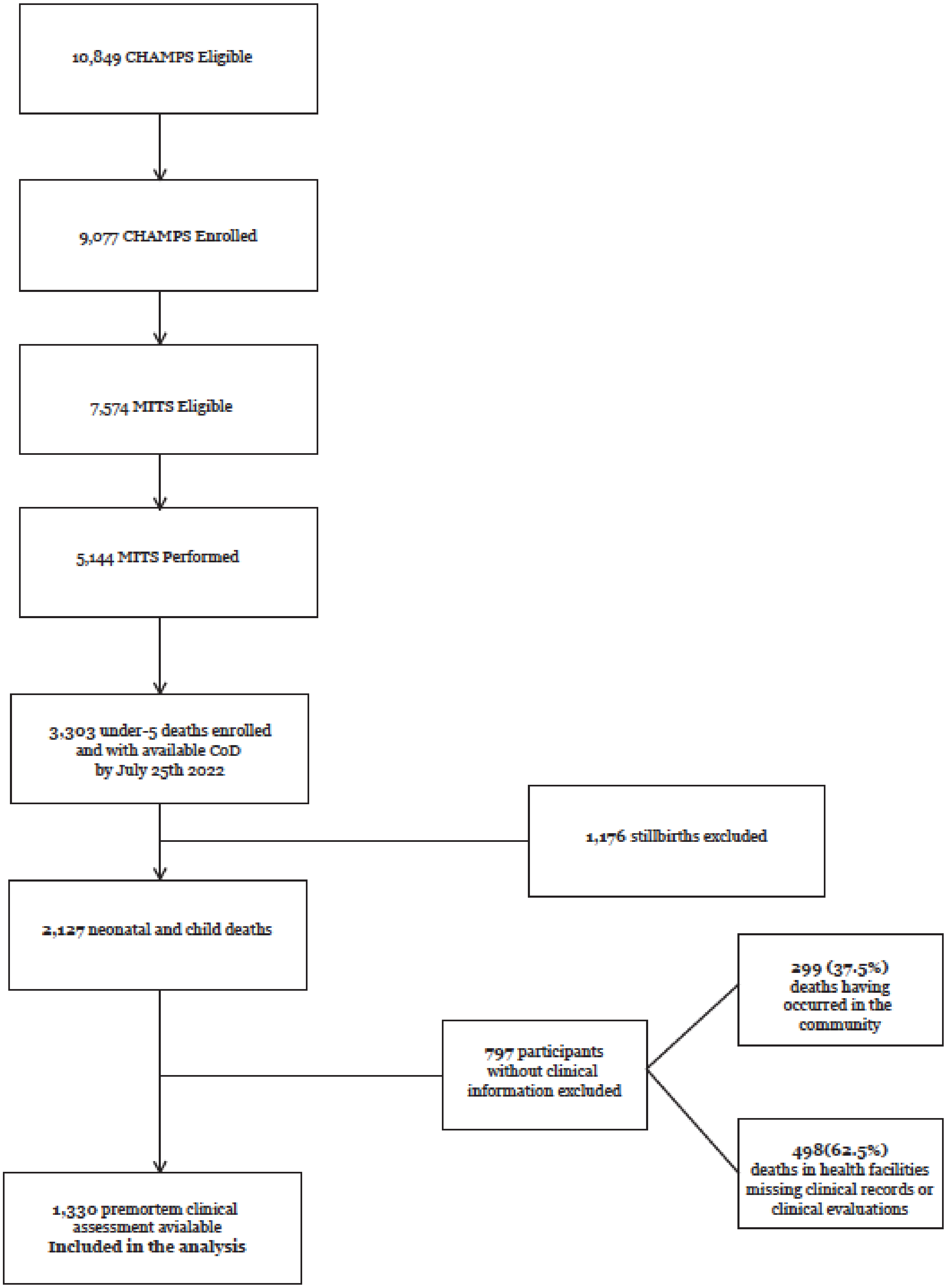

By the time of the analysis, 10849 deaths were notified in the study areas. Of those, 9077 were enrolled in CHAMPS, 7574 were MITS eligible, and 5144 MITS were performed. Decode panels had 3303 cases decodified by July 2022, and those participants were assessed for this analysis. After excluding stillbirths, 2127 neonatal deaths were included, and of those 1330 had a neurological evaluation recorded in their examination prior to death. Among patients without neurological evaluations available, 37.5% were deaths that occurred in the community without medical examination, and 62.5% occurred in health facilities but did not have a neurological exam recorded.

eTable 4. Participants Enrolled per Year and Country

	2016		***2017***		***2018***		***2019***		***2020***		***2021***	
	Deaths with MITS and DeCoDe	With neurological evaluation and included in analysis	Deaths with MITS and DeCoDe	With neurological evaluation and included in analysis	Deaths with MITS and DeCoDe	With neurological evaluation and included in analysis	Deaths with MITS and DeCoDe	With neurological evaluation and included in analysis	Deaths with MITS and DeCoDe	With neurological evaluation and included in analysis	Deaths with MITS and DeCoDe	With neurological evaluation and included in analysis
Bangladesh	0	0	5	4 (80%)	20	18 (90%)	56	46 (82%)	28	22 (79%)	72	67 (93%)
Ethiopia	0	0	0	0	0	0	28	14 (50%)	46	28 (61%)	53	26 (49%)
Kenya	0	0	34	26 (76%)	98	58 (59%)	138	56 (41%)	67	45 (67%)	72	46 (64%)
Mali	0	0	20	15 (75%)	44	26 (59%)	43	21 (49%)	15	11 (73%)	4	4 (100%)
Mozambique	0	0	54	42 (78%)	44	31 (70%)	135	100 (74%)	142	88 (62%)	22	17 (77%)
Sierra Leone	0	0	0	0	0	0	125	86 (69%)	33	26 (79%)	80	42 (53%)
South Africa	1	1 (100%)	283	193 (68%)	130	72 (55%)	70	27 (39%)	84	41 (49%)	81	31 (38%)
**Total**	***1***	***1 (100%)***	***396***	***280 (71%)***	***336***	***205 (61%)***	***595***	***350 (59%)***	***373***	***261 (70%)***	***384***	***233 (61%)***

Total enrolled children per year and country, and proportion of children with neurological evaluations available and, therefore, included in the analysis. Of note, for year 2022 we don't have granular data by country and site, as the analysis was performed until July 25th and not during the entire year.

© 2024 Ajanovic S et al. *JAMA Network Open.*

eTable 5. Sensitivity and Specificity of Case Definitions for Suspected Meningitis, Cerebral Malaria, and Neonatal Hypoxic Events (Including Both Perinatal Asphyxia and Neonatal Encephalopathy)

	TP	Cases	Sensitivity (95% CI)	Specificity (95% CI)	PPV (95% CI)	NPV (95% CI)
**Suspected meningitis**						
**Fever + one of the following: meningeal signs, altered consciousness, seizures**	16	46	34·8 % (CI 21·35 - 50·25%)	61·7% (CI 57·2% - 66·05%)	7·96% (CI 5·42 - 11·55%)	90·85% (CI 88·83 to 92·54%)
**In neonates: Fever or one of the following: bulging fontanel, altered consciousness, seizures.**	55	89	61·8% (CI 50·89% - 71·9%)	48·26% (CI 45·51% - 49·25%)	12·42% (CI 10·62%- 14·7%)	90·5% (87·85% - 92·63%)
**Cerebral malaria**						
**Loss of consciousness + positive malaria test**	10	50	20% (CI 10·3% - 33·72%)	97·61% (CI 94·87% - 99·12%)	62·5% (38·82% to 81·4%)	97·47 % (85·96% to 87·57%)
**Neonatal hypoxia**						
**Altered mental state or seizures**	45	300	15% (CI 11·16% - 19·55%)	91·22% (88·39% - 93·55%)	50·56% (40·91% - 60·17%)	64·19% (62·92% - 65·43%)

TP: true positive, meaning number of cases matching suspected case definition and + ICD-10 diagnosis, Cases: number of cases with a final ICD-10 diagnosis. CI: confidence interval. PPV: positive predictive value, NPV: negative predictive value. Suspected meningitis has been calculated only for those with available information for "fever".

Suspected cerebral malaria calculated only for those with a malaria test result available (n=301). Suspected neonatal consequences of hypoxic events have only been calculated among neonates (n=801).

© 2024 Ajanovic S et al. *JAMA Network Open.*

eTable 6. Sensitivity and Specificity of Each Sign and Combination of Signs Associated With the Most Common Neurological Diagnoses

a) Sensitivity

Sensitivity (%)															
Neonatal Hypoxic events				Neonatal meningoencephalitis				Infants and Children malaria				Infants and Children meningoencephalitis			
1 sign	2 signs	3 signs	4 signs	1 sign	2 signs	3 signs	4 signs	1 sign	2 signs	3 signs	4 signs	1 sign	2 signs	3 signs	4 signs
SZ	SZ,LC	SZ,LC,AMS	SZ,LC,AMS,MN	SZ	SZ,LC	SZ,LC,AMS	SZ,LC,AMS,MN	SZ	SZ,LC	SZ,LC,AMS	SZ,LC,AMS,MN	SZ	SZ,LC	SZ,LC,AMS	SZ,LC,AMS,MN
31,8	2,4	0,7		34,8	1,1	1,1		51,5	16,2	5,9		46,7	6,7	4,4	
LC	SZ,AMS	SZ,LC,MN	SZ,LC,AMS,F	LC	SZ,AMS	SZ,LC,MN	SZ,LC,AMS,F	LC	SZ,AMS	SZ,LC,MN	SZ,LC,AMS,F	LC	SZ,AMS	SZ,LC,MN	SZ,LC,AMS,F
6,6	5,6			3,4	2,2		1,1	20,6	13,2		4,4	13,3	8,9		2,2
AMS	SZ,MN	SZ,LC,F	SZ,LC,MN,F	AMS	SZ,MN	SZ,LC,F	SZ,LC,MN,F	AMS	SZ,MN	SZ,LC,F	SZ,LC,MN,F	AMS	SZ,MN	SZ,LC,F	SZ,LC,MN,F
19,6		0,3		10,1	2,2	1,1		17,6	2,9			17,8	2,2	4,4	
MN	SZ,F	SZ,AMS,MN	SZ,AMS,MN,F	MN	SZ,F	SZ,AMS,MN	SZ,AMS,MN,F	MN	SZ,F	SZ,AMS,MN	SZ,AMS,MN,F	MN	SZ,F	SZ,AMS,MN	SZ,AMS,MN,F
0,7	2,4			2,2	4,5			2,9	25	8,8	1,5	4,4	15,6		
F	LC,AMS	SZ,AMS,F	LC,AMS,MN,F	F	LC,AMS	SZ,AMS,F	LC,AMS,MN,F	F	LC,AMS	SZ,AMS,F	LC,AMS,MN,F	F	LC,AMS	SZ,AMS,F	LC,AMS,MN,F
4,5	3,5	0,7		11,2	2,2	1,1		48,5	7,4	8,8		31,1	8,9	6,7	
	LC,MN	SZ,MN,F			LC,MN	SZ,MN,F			LC,MN	SZ,MN,F			LC,MN	SZ,MN,F	
										1,2				2,2	
	LC,F	LC,AMS,MN			LC,F	LC,AMS,MN			LC,F	LC,AMS,MN			LC,F	LC,AMS,MN	
	0,3				1,1				10,3				8,9		
	AMS,MN	LC,AMS,F			AMS,MN	LC,AMS,F			AMS,MN	LC,AMS,F			AMS,MN	LC,AMS,F	
						1,1			1,5	4,4				4,4	
	AMS,F	LC,MN,F			AMS,F	LC,MN,F			AMS,F	LC,MN,F			AMS,F	LC,MN,F	
	1				1,1				8,8				8,9		
	MN,F	AMS,MN,F			MN,F	AMS,MN,F			MN,F	AMS,MN,F			MN,F	AMS,MN,F	
	0,3				1,1				1,5				2,2		

© 2024 Ajanovic S et al. *JAMA Network Open.*

b) Specificity

Specificity (%)															
**Neonatal Hypoxic events**				**Neonatal Meningitis**				**Infants and Children malaria**				**Infants and Children meningitis**			
**1 sign**	**2 signs**	**3 signs**	**4 signs**	**1 sign**	**2 signs**	**3 signs**	**4 signs**	**1 sign**	**2 signs**	**3 signs**	**4 signs**	**1 sign**	**2 signs**	**3 signs**	**4 signs**
SZ	SZ,LC	SZ,LC,AMS	SZ,LC,AMS,MN	SZ	SZ,LC	SZ,LC,AMS	SZ,LC,AMS,MN	SZ	SZ,LC	SZ,LC,AMS	SZ,LC,AMS,MN	SZ	SZ,LC	SZ,LC,AMS	SZ,LC,AMS,MN
82,2	98,8	99		78,7	98,3	99,2		74,8	94,8	97,6		73,6	93,8	97,5	
LC	SZ,AMS	SZ,LC,MN	SZ,LC,AMS,F	LC	SZ,AMS	SZ,LC,MN	SZ,LC,AMS,F	LC	SZ,AMS	SZ,LC,MN	SZ,LC,AMS,F	LC	SZ,AMS	SZ,LC,MN	SZ,LC,AMS,F
96,7	97,9			95,4	96,5		100	89,6	94,6		98,7	88,8	94,6		98,3
AMS	SZ,MN	SZ,LC,F	SZ,LC,MN,F	AMS	SZ,MN	SZ,LC,F	SZ,LC,MN,F	AMS	SZ,MN	SZ,LC,F	SZ,LC,MN,F	AMS	SZ,MN	SZ,LC,F	SZ,LC,MN,F
88,4		99,6		85	100	99,7		83,3	98,5			83,7	98,6	96,7	
MN	SZ,F	SZ,AMS,MN	SZ,AMS,MN,F	MN	SZ,F	SZ,AMS,MN	SZ,AMS,MN,F	MN	SZ,F	SZ,AMS,MN	SZ,AMS,MN,F	MN	SZ,F	SZ,AMS,MN	SZ,AMS,MN,F
98,3	96,3			98,7	96,9			97,2	90,2	97,2	99,8	97,5	88,6		
F	LC,AMS	SZ,AMS,F	LC,AMS,MN,F	F	LC,AMS	SZ,AMS,F	LC,AMS,MN,F	F	LC,AMS	SZ,AMS,F	LC,AMS,MN,F	F	LC,AMS	SZ,AMS,F	LC,AMS,MN,F
89,5	98,1	99,2		92	97,5	99,3		68,2	95,7	97,4		64,9	96,3	97,1	
	LC,MN	SZ,MN,F			LC,MN	SZ,MN,F			LC,MN	SZ,MN,F			LC,MN	SZ,MN,F	
										99,6				99,6	
	LC,F	LC,AMS,MN			LC,F	LC,AMS,MN			LC,F	LC,AMS,MN			LC,F	LC,AMS,MN	
	99,4				100				95,4				95,2		
	AMS,MN	LC,AMS,F			AMS,MN	LC,AMS,F			AMS,MN	LC,AMS,F			AMS,MN	LC,AMS,F	
						99,9			99,1	97,8				97,7	
	AMS,F	LC,MN,F			AMS,F	LC,MN,F			AMS,F	LC,MN,F			AMS,F	LC,MN,F	
	98,3				98,5				93,7				93,8		
	MN,F	AMS,MN,F			MN,F	AMS,MN,F			MN,F	AMS,MN,F			MN,F	AMS,MN,F	
	99,6				99,7				98,7				98,8		

Among neonates, we have evaluated hypoxic events and meningitis / encephalitis, and in infants and children meningitis / encephalitis and cerebral malaria, as the most common differential diagnoses by age groups. SZ: seizures, LC: loss of consciousness, AMS: altered mental state, MN: meningeal signs, F: fever. The sensitivity and specificity values are shown in %. The grading color is concordant to their % value.

© 2024 Ajanovic S et al. *JAMA Network Open.*

eTable 7. Characteristics of Participants Who Had a Lumbar Puncture Performed Prior to Death (n = 95)

		Clinical presentation						Results of premortem Lumbar Puncture culture		Diagnosis after Decode Panel								
Participant	Age category	Any neurological sign?	Seizures at presentation	Seizures during hospitalization	Loss of consciousness	Altered mental state	Meningeal signs	Result	Pathogen	Any neurological diagnosis?	Perinatal asphyxia	Neonatal encephalopathy	Meningoencephalitis	Cerebral malara	Other neurological diagnoses	ICD Code	Diagnosis	Etiology if identified
1	I	+				+		**Positive**	***Neisseria Meningitidis***	+			+			**A39·2**	**Meningitis/Encephalitis**	***Neisseria meningitidis***
2	N	+	+			+		**Positive**	***Escherchia Coli***	+			+			**G00·8**	**Meningitis/Encephalitis**	***Escherchia coli***
3	N	+		+				**Positive**	***Acinetobacter Baumanii***	+			+			**G00·8**	**Meningitis/Encephalitis**	***Acinetobacter baumannii***
4	N	+		+				**Positive**	***Acinetobacter Baumanii***	+			+			**G00·8**	**Meningitis/Encephalitis**	***Acinetobacter baumannii***
5	N	+			+			**Positive**	***Acinetobacter Baumanii***	+			+			**G00·8**	**Meningitis/Encephalitis**	***Acinetobacter baumannii***
6	N	+		+				**Positive**	***Acinetobacter Baumanii***	+			+			**G03·9**	**Meningitis/Encephalitis**	
7	I	+				+		Negative		+			+			**A85.+**	**Meningitis/Encephalitis**	**Adenovirus**
8	N							NA		+			+			**B37·5**	**Meningitis/Encephalitis**	***Candida albicans***
9	I	+		+				NA		+			+			**B37·5**	**Meningitis/Encephalitis**	***Candida parapsilosis***
10	I	+		+				NA		+			+			**B37·5**	**Meningitis/Encephalitis**	***Candida albicans***
11	I	+		+		+		NA		+	+		+			**G00·3**	**Meningitis/Encephalitis**	***Methicillinresistant Staphylococcus aureus***
12	N	+		+				NA		+			+			**G00·8**	**Meningitis/Encephalitis**	***Acinetobacter baumannii***
13	N	+				+		NA		+			+			**G00·8**	**Meningitis/Encephalitis**	***Acinetobacter baumannii***
14	C	+	+	+	+			NA		+			+			**G00·8**	**Meningitis/Encephalitis**	***Streptococcus pneumoniae***
15	N	+		+				NA		+	+	+	+			**G00·8**	**Meningitis/Encephalitis**	***Klebsiella pneumoniae***
16	N	+		+				NA		+			+			**G00·8**	**Meningitis/Encephalitis**	***Acinetobacter baumannii***
17	N	+		+				NA		+			+			**G00·8**	**Meningitis/Encephalitis**	***Acinetobacter baumannii***
18	N	+		+				NA		+			+			**G00·8**	**Meningitis/Encephalitis**	***Acinetobacter baumannii***
19	N							NA		+			+			**G00·8**	**Meningitis/Encephalitis**	***Acinetobacter baumannii***
20	N							NA		+			+			**G00·8**	**Meningitis/Encephalitis**	***Klebsiella pneumoniae***
21	C	+	+	+		+		NA		+			+			**G03·9**	**Meningitis/Encephalitis**	
22	N	+		+				NA		+	+	+	+			**G03·9**	**Meningitis/Encephalitis**	
23	I	+				+		NA		+			+			**G03·9**	**Meningitis/Encephalitis**	***Pneumocystis jirovecii***
24	I	+		+				NA		+			+			**G04·8**	**Meningitis/Encephalitis**	***Candida albicans***
25	C	+		+		+		NA		+			+			**G04·8**	**Meningitis/Encephalitis**	
26	C	+	+	+		+		Negative		+				+		B50·0	Cerebral malaria	*Plasmodium falciparum*
27	I							Negative		+		+						
28	N	+			+			Negative		+	+							
29	N	+		+				Negative		+	+	+						
30	N	+		+				Negative		+	+	+						
31	C	+	+		+			NA		+								
32	N	+	+	+		+	+	NA		+				+		B50·0	Cerebral malaria	*Plasmodium falciparum*
33	C	+	+	+				NA		+					+	G41·9	Status epillepticus	
34	N	+			+			NA		+					+	G91·9	Hydrocephalus	
35	N	+	+	+				NA		+	+	+						
36	N	+		+				NA		+	+	+				P36·8	Sepsis	*Streptococcus viridans*
37	N	+		+				NA		+	+					P36·4	Sepsis	*Escherichia coli*
38	N	+		+				NA		+	+	+				P36·4	Sepsis	*Escherichia coli*
39	N	+		+		+		NA		+						P36·8	Sepsis	*Klebsiella pneumoniae*
40	N	+		+				NA		+	+					P36·8	Sepsis	*Acinetobacter baumannii*
41	N	+		+	+			NA		+	+	+						
42	N	+		+				NA		+	+	+						

© 2024 Ajanovic S et al. *JAMA Network Open.*

43	I	+		+		+		Negative						
44	N	+		+				Contam.	Bacillus spp	+	+	G40·9	Epilepsy	
45	N							**Positive**	*Acinetobacter Baumanii*			P36·8	Sepsis	*Acinetobacter baumannii*
46	I							**Positive**	Fungi (unspecified)			P37·5	Neonatal complications	*Candida parapsilosis*
47	I	+	+	+		+		Negative				A41·5	Sepsis	*Klebsiella pneumoniae*
48	I	+	+	+				Negative				A41·5	Sepsis	*Klebsiella pneumoniae*
49	N	+				+		Negative				A41·9	Sepsis	Polimicrobian
50	N							Negative				B37·7	Sepsis	*Candida albicans*
51	N	+				+		Negative				P36·8	Sepsis	*Acinetobacter baumannii*
52	N	+	+	+				Negative				P36·8	Sepsis	*Acinetobacter baumannii*
53	I							Negative				P36·8	Sepsis	*Acinetobacter baumannii*
54	C	+		+			+	Negative		+	+	T60·0	Poisoning	
55	C	+					+	Negative						
56	I	+	+	+	+	+		Negative						
57	I							Negative						
58	C							NA						
59	I	+			+		+	NA				A40	Sepsis	*Streptococcus pneumoniae*
60	I	+		+				NA				A41·0	Sepsis	*Staphylococcus aureus*
61	I							NA				A41·0	Sepsis	*Staphylococcus aureus*
62	C	+				+		NA				A41·5	Sepsis	*Escherichia coli*
63	I	+				+		NA				A41·5	Sepsis	*Pseudomonas aerugisa*
64	I	+		+				NA				A41·5	Sepsis	*Acinetobacter baumannii*
65	I							NA				A41·5	Sepsis	*Escherichia coli*
66	I	+				+		NA				A41·8	Sepsis	*Klebsiella pneumoniae*
67	C	+				+		NA				A41·9	Sepsis	
68	I	+	+	+				NA				A41·9	Sepsis	
69	N							NA		+	+	G06·0	Intracraneal abscess	
70	N	+				+		NA						
71	N							NA						
72	N							NA				P36·0	Sepsis	*Streptococcus agalactiae*
73	N	+		+				NA				P36·8	Sepsis	*Candida glabrata*
74	N	+		+				NA				P36·8	Sepsis	*Acinetobacter baumannii*
75	N							NA				P36·8	Sepsis	*Acinetobacter baumannii*
76	N							NA				P36·8	Sepsis	*Acinetobacter baumannii*
77	N							NA						
78	C	+	+		+			NA						
79	C	+				+		NA						
80	C	+				+		NA						
81	C	+	+	+				NA						
82	I	+	+	+		+		NA						
83	C	+	+	+		+		NA						
84	I	+		+				NA						
85	N	+				+		NA						
86	I	+			+			NA						
87	I	+				+		NA						
88	I	+			+			NA						
89	C	+				+		NA						
90	C	+				+		NA						
91	I	+				+		NA						
92	I							NA						
93	I	+				+		Contam.	CNS and *S.viridans*			A40·3	Sepsis	*Streptococcus pneumoniae*
94	N	+				+		Contam.	Gram negative bacillus			P36·8	Sepsis	*Acinetobacter baumannii*
95	N	+	+	+				Contam.	CNS			P36·8	Sepsis	*Klebsiella pneumoniae*

© 2024 Ajanovic S et al. *JAMA Network Open.*

In age category: N= neonatal death, I= infant, C= child. Positive signs represent the presence of specific neurological signs or symptoms. Patients who have any kind of neurological sign (+) that is not included among the 4 most common ones, presented less representative neurological conditions (abnormal position, focal neurological deficit, decerebration, decortication). NA=Not available. Contam = Culture with the detection of a contaminant pathogen. CNS= coagulase negative staphylococcus. ICD-10= International Classification of Diseases, 10th version (WHO).

© 2024 Ajanovic S et al. *JAMA Network Open.*

eTable 8. Presence of Neurological Symptoms Across All Causes of Death Included in the Chain of Events Leading to Death

Cause of death	Any neurological symptom N = 727 n (%)	Seizures at presentation N = 206 n (%)	Seizures during hospitalization N = 301 n (%)	Loss of consciousness N = 170 n (%)	Altered mental status N = 472 n (%)	Meningeal signs N = 30 n (%)	Perinatal asphyxia N = 287 n (%)	Neonatal encephalopathy N = 66 n (%)	Meningo-encephalitis N = 135 n (%)	Cerebral Malaria N = 68 n (%)
Anemias	46 (6.3)	25 (12.1)	21 (7.0)	19 (11.2)	27 (5.7)	1 (3.3)	1 (0.3)	0 (0)	5 (3.7)	28 (41.2)
Birth trauma	2 (0.3)	1 (0.5)	1 (0.3)	0 (0)	1 (0.2)	0 (0)	3 (1.0)	0 (0)	0 (0)	0 (0)
Cancer	3 (0.4)	0 (0)	1 (0.3)	1 (0.6)	3 (0.6)	0 (0)	0 (0)	0 (0)	0 (0)	0 (0)
Cesarean delivery	1 (0.1)	0 (0)	0 (0)	0 (0)	0 (0)	1 (3.3)	0 (0)	0 (0)	0 (0)	0 (0)
Chorioamnionitis and membrane complications	2 (0.3)	0 (0)	0 (0)	0 (0)	2 (0.4)	0 (0)	1 (0.3)	0 (0)	0 (0)	0 (0)
Congenital birth defects	67 (9.2)	16 (7.8)	27 (9.0)	9 (5.3)	37 (7.8)	6 (20.0)	17 (5.9)	1 (1.5)	8 (5.9)	0 (0)
Congenital infection	36 (5.0)	2 (1.0)	20 (6.6)	6 (3.5)	25 (5.3)	1 (3.3)	17 (5.9)	4 (6.1)	8 (5.9)	0 (0)
Diabetes	1 (0.1)	1 (0.5)	1 (0.3)	1 (0.6)	1 (0.2)	0 (0)	0 (0)	0 (0)	0 (0)	0 (0)
Diarrheal Diseases	56 (7.7)	15 (7.3)	20 (6.6)	19 (11.2)	46 (9.7)	2 (6.7)	0 (0)	0 (0)	4 (3.0)	0 (0)
Epilepsy	4 (0.6)	3 (1.5)	4 (1.3)	1 (0.6)	1 (0.2)	0 (0)	0 (0)	0 (0)	0 (0)	0 (0)
Heart Diseases	1 (0.1)	0 (0)	0 (0)	0 (0)	1 (0.2)	0 (0)	0 (0)	0 (0)	0 (0)	0 (0)
HIV	39 (5.4)	8 (3.9)	10 (3.3)	8 (4.7)	31 (6.6)	1 (3.3)	0 (0)	0 (0)	3 (2.2)	4 (5.9)
Injury	6 (0.8)	4 (1.9)	4 (1.3)	1 (0.6)	2 (0.4)	0 (0)	0 (0)	0 (0)	1 (0.7)	0 (0)
Kidney Disease	7 (1.0)	0 (0)	5 (1.7)	0 (0)	3 (0.6)	0 (0)	0 (0)	0 (0)	2 (1.5)	0 (0)
Liver disease	12 (1.7)	2 (1.0)	3 (1.0)	3 (1.8)	10 (2.1)	0 (0)	0 (0)	0 (0)	4 (3.0)	0 (0)
Lower respiratory infections	203 (27.9)	55 (26.7)	86 (28.6)	38 (22.4)	123 (26.1)	8 (26.7)	20 (7.0)	7 (10.6)	69 (51.1)	4 (5.9)
Malaria	73 (10.0)	43 (20.9)	35 (11.6)	27 (15.9)	39 (8.3)	2 (6.7)	0 (0)	0 (0)	0 (0)	68 (100.0)
Malnutrition	80 (11.0)	33 (16.0)	30 (10.0)	25 (14.7)	47 (10.0)	5 (16.7)	0 (0)	0 (0)	8 (5.9)	12 (17.6)
Maternal hypertension	1 (0.1)	0 (0)	0 (0)	0 (0)	1 (0.2)	0 (0)	0 (0)	0 (0)	0 (0)	0 (0)
Maternal infection	1 (0.1)	0 (0)	0 (0)	1 (0.6)	1 (0.2)	0 (0)	0 (0)	0 (0)	0 (0)	0 (0)
Measles	1 (0.1)	0 (0)	1 (0.3)	0 (0)	1 (0.2)	0 (0)	0 (0)	0 (0)	0 (0)	0 (0)
Meningitis/Encephalitis	86 (11.8)	20 (9.7)	49 (16.3)	15 (8.8)	46 (9.7)	4 (13.3)	11 (3.8)	4 (6.1)	134 (99.3)	0 (0)
Neonatal aspiration syndromes	24 (3.3)	3 (1.5)	12 (4.0)	2 (1.2)	18 (3.8)	0 (0)	21 (7.3)	5 (7.6)	7 (5.2)	0 (0)
Neonatal encephalopathy	57 (7.8)	21 (10.2)	44 (14.6)	14 (8.2)	33 (7.0)	0 (0)	50 (17.4)	66 (100.0)	4 (3.0)	0 (0)
Neonatal preterm birth complications	150 (20.6)	11 (5.3)	55 (18.3)	25 (14.7)	100 (21.2)	2 (6.7)	62 (21.6)	4 (6.1)	64 (47.4)	0 (0)
Other	15 (2.1)	1 (0.5)	4 (1.3)	5 (2.9)	11 (2.3)	1 (3.3)	2 (0.7)	2 (3.0)	14 (10.4)	1 (1.5)
Other disorders of fluid, electrolyte and acid-base balance	7 (1.0)	2 (1.0)	5 (1.7)	0 (0)	5 (1.1)	0 (0)	0 (0)	0 (0)	2 (1.5)	0 (0)
Other endocrine, metabolic, blood, and immune disorders	13 (1.8)	3 (1.5)	2 (0.7)	3 (1.8)	13 (2.8)	0 (0)	0 (0)	0 (0)	6 (4.4)	2 (2.9)
Other immunodeficiencies	2 (0.3)	0 (0)	0 (0)	0 (0)	2 (0.4)	0 (0)	0 (0)	0 (0)	0 (0)	0 (0)
Other infections	28 (3.9)	8 (3.9)	10 (3.3)	5 (2.9)	19 (4.0)	1 (3.3)	0 (0)	1 (1.5)	10 (7.4)	0 (0)
Other neonatal disorders	67 (9.2)	12 (5.8)	38 (12.6)	10 (5.9)	35 (7.4)	1 (3.3)	26 (9.1)	2 (3.0)	30 (22.2)	0 (0)
Other neurological disorders	20 (2.8)	12 (5.8)	15 (5.0)	6 (3.5)	11 (2.3)	2 (6.7)	5 (1.7)	1 (1.5)	3 (2.2)	0 (0)
Other respiratory disease	46 (6.3)	19 (9.2)	16 (5.3)	15 (8.8)	29 (6.1)	3 (10.0)	2 (0.7)	1 (1.5)	4 (3.0)	6 (8.8)
Other skin and subcutaneous diseases	2 (0.3)	0 (0)	1 (0.3)	0 (0)	1 (0.2)	0 (0)	0 (0)	0 (0)	0 (0)	0 (0)
Paralytic ileus and intestinal obstruction	6 (0.8)	3 (1.5)	1 (0.3)	3 (1.8)	4 (0.8)	0 (0)	0 (0)	0 (0)	2 (1.5)	0 (0)

© 2024 Ajanovic S et al. *JAMA Network Open.*

Perinatal asphyxia/hypoxia	172 (23.7)	39 (18.9)	86 (28.6)	40 (23.5)	112 (23.7)	2 (6.7)	276 (96.2)	50 (75.8)	12 (8.9)	0 (0)
Placental complications	1 (0.1)	0 (0)	1 (0.3)	0 (0)	0 (0)	0 (0)	0 (0)	0 (0)	0 (0)	0 (0)
Poisoning	5 (0.7)	2 (1.0)	2 (0.7)	1 (0.6)	3 (0.6)	1 (3.3)	0 (0)	0 (0)	1 (0.7)	0 (0)
Sepsis	275 (37.8)	58 (28.2)	124 (41.2)	55 (32.4)	174 (36.9)	10 (33.3)	52 (18.1)	9 (13.6)	106 (78.5)	8 (11.8)
Sickle cell disorders	2 (0.3)	1 (0.5)	1 (0.3)	1 (0.6)	1 (0.2)	0 (0)	0 (0)	0 (0)	0 (0)	0 (0)
Syphilis	3 (0.4)	0 (0)	1 (0.3)	1 (0.6)	3 (0.6)	0 (0)	0 (0)	0 (0)	0 (0)	0 (0)
Tuberculosis	8 (1.1)	4 (1.9)	4 (1.3)	1 (0.6)	7 (1.5)	1 (3.3)	0 (0)	0 (0)	0 (0)	1 (1.5)
Undetermined	7 (1.0)	3 (1.5)	2 (0.7)	1 (0.6)	3 (0.6)	0 (0)	0 (0)	0 (0)	0 (0)	0 (0)
Upper respiratory infections	1 (0.1)	0 (0)	0 (0)	0 (0)	1 (0.2)	0 (0)	0 (0)	0 (0)	0 (0)	0 (0)

For each death we have multiple diagnoses included in the chain of events leading to death, therefore, some diseases that are frequently associated or concomitant to neurological diagnoses, are highly represented in the table. The three groups of neurological diagnoses have been included in the table for its comparison.

eTable 9. Presence of Neurological Symptoms Across Causes of Death, Grouped by Syndrome

	**Any neurological symptom**	**Seizures at presentation**	**Seizures during hospitalization**	**Loss of consciousness**	**Altered mental status**	**Meningeal signs**	**Perinatal asphyxia**	**Neonatal encephalopathy**	**Meningo-encephalitis**	**Cerebral Malaria**
	N = 727	N = 206	N = 301	N = 170	N = 472	N = 30	N = 287	N = 66	N = 135	N = 68
**Cause of death**	n (%)	n (%)	n (%)	n (%)	n (%)	n (%)	n (%)	n (%)	n (%)	n (%)
Anemias	46 (6.3)	25 (12.1)	21 (7.0)	19 (11.2)	27 (5.7)	1 (3.3)	1 (0.3)	0 (0)	5 (3.7)	28 (41.2)
Congenital birth defects	67 (9.2)	16 (7.8)	27 (9.0)	9 (5.3)	37 (7.8)	6 (20.0)	18 (6.3)	1 (1.5)	8 (5.9)	0 (0)
Diarrheal Diseases	56 (7.7)	15 (7.3)	20 (6.6)	19 (11.2)	46 (9.7)	2 (6.7)	0 (0)	0 (0)	4 (3.0)	0 (0)
HIV	39 (5.4)	8 (3.9)	10 (3.3)	8 (4.7)	31 (6.6)	1 (3.3)	0 (0)	0 (0)	3 (2.2)	4 (5.9)
Lower respiratory infections	203 (27.9)	55 (26.7)	86 (28.6)	38 (22.4)	123 (26.1)	8 (26.7)	20 (7.0)	7 (10.6)	69 (51.1)	4 (5.9)
Malaria	73 (10.0)	43 (20.9)	35 (11.6)	27 (15.9)	39 (8.3)	2 (6.7)	0 (0)	0 (0)	0 (0)	68 (100.0)
Malnutrition	80 (11.0)	33 (16.0)	30 (10.0)	25 (14.7)	47 (10.0)	5 (16.7)	0 (0)	0 (0)	8 (5.9)	12 (17.6)
Meningitis/Encephalitis	86 (11.8)	20 (9.7)	49 (16.3)	15 (8.8)	46 (9.7)	4 (13.3)	11 (3.8)	4 (6.1)	134 (99.3)	0 (0)
Neonatal preterm birth complications	330 (45.4)	57 (27.7)	147 (48.8)	66 (38.8)	217 (46.0)	6 (20.0)	287 (100.0)	66 (100.0)	81 (60.0)	0 (0)
Sepsis	276 (38.0)	58 (28.2)	124 (41.2)	55 (32.4)	175 (37.1)	10 (33.3)	55 (19.2)	9 (13.6)	106 (78.5)	8 (11.8)
Other	209 (28.7)	64 (31.1)	97 (32.2)	46 (27.1)	132 (28.0)	9 (30.0)	34 (11.8)	4 (6.1)	59 (43.7)	8 (11.8)
Undetermined	7 (1.0)	3 (1.5)	2 (0.7)	1 (0.6)	3 (0.6)	0 (0)	0 (0)	0 (0)	0 (0)	0 (0)

Of note, for each death we have multiple diagnoses included in the chain of events leading to death, therefore, some diseases that are frequently associated or concomitant to neurological diagnoses, are highly represented in the table. The three groups of neurological diagnoses have been included in the table for its comparison.

© 2024 Ajanovic S et al. *JAMA Network Open.*

eTable 10. Signs and Management Decisions Described by Final Neurological Diagnosis (After DeCoDe Panel Discussions)

	Perinatal asphyxia		Neonatal encephalopathy		Meningoencephalitis		Cerebral Malaria	
	**N**	**%**	**N**	**%**	**N**	**%**	**N**	**%**
Any signs or symptoms	174	60·6%	57	86·4%	87	64·4%	50	73·5
Seizures at presentation	41	22·3%	21	56·8%	20	29·4	34	70·8%
Seizures during hospitalization	87	38·3%	44	77·2%	49	55·1%	30	63·8%
Loss of consciousness	40	19·5%	14	19·2%	16	15·0%	20	40·8%
Altered mental status	113	55·9%	33	70·2%	47	44·3%	26	52·0%
Meningeal signs	2	3·3%	0	0·0%	4	11·8%	2	22·2%
Lumbar puncture	13	27·1%	10	62·5%	25	50%	2	8·3%
Antibiotic treatment	222	77·4%	51	77·3%	113	83·7	51	75·0%
Antimalarial treatment	6	2·1%	0	0·0%	6	4·4%	49	72·1%

Complete case definitions have been used for each variable.

eTable 11. Neurological Signs Present in Children With Cerebral Malaria and No-Cerebral Malaria Listed in the Chain of Events Leading to Death

Participants with malaria diagnoses included in the chain of events leading to death

	Total	Cerebral malaria	Non-cerebral malaria	Chi-square p-value
	118	68	50	
Seizures	60 (50.8%)	36 (52.9%)	24 (48%)	0.731
Loss of consciousness	26 (22%)	20 (29.4%)	6 (12%)	0.042
Meningeal signs	2 (1.6%)	2 (2.9%)	0 (0%)	0.616
Altered mental state	20 (16.9%)	12 (17.6%)	8 (16%)	1.0

© 2024 Ajanovic S et al. *JAMA Network Open.*

eTable 12. Malaria Testing in the Cohort, and Prescription of Antimalarial and Antimicrobial Treatment

MALARIA TEST	TOTAL	ANTIMALARIAL TREATMENT	ANTIBIOTIC TREATMENT
**PERFORMED**	285	108 (37.9%)	258 (90.5%)
POSITIVE	88	85 (96.5%)	79 (89.8%)
NEGATIVE	197	23 (11.7%)	179 (90.1%)
**NOT PERFORMED OR NOT RECORDED**	1045	34 (3.3%)	614 (58.8%)

© 2024 Ajanovic S et al. *JAMA Network Open.*
