# Supplementary material for: Neurological Symptoms and Cause of Death Among Young Children in Low- and Middle-Income Countries
Source: JAMA Netw Open. 2024 Sep 3;7(9):e2431512. doi: 10.1001/jamanetworkopen.2024.31512 (PMC11372484; doi:10.1001/jamanetworkopen.2024.31512)
Supplement: Supplement 2. — Nonauthor Collaborators [file jamanetwopen-e2431512-s002.pdf]

**Supplement 2.** Nonauthor Collaborators
*First name, last name, and suffix (if applicable) are required and will appear in PubMed.

| *Group Name: Child Health and Mortality Prevention Surveillance (CHAMPS) Consortium | | | | | | | |
|---|---|---|---|---|---|---|---|
| ***First Name and Middle Initial(s)** | ***Last Name** | ***Suffix (eg, Jr, III)** | Academic Degrees | Institution | Location (city, state/province, country) | Role or Contribution, eg, chair, principal investigator | Group (if more than 1 Group listed in the byline) and/or Subgroup (eg, Steering Committee) |
| Fatima | Solomon | | MD | South African Medical Research Council Vaccines and Infectious Diseases Analytics Research Unit, University of the Witwatersrand, Johannesburg, South Africa | South Africa | Data collection | |
| Gillian | Sorour | | MD | Wits Health Consortium | South Africa | Data collection | |
| Hennie | Lombaard | | MD | University of Witwatersrand, Johannesburg, South Africa | South Africa | Data collection | |
| Jeannette | Wadula | | MD | National Health Laboratory Service, Department of Microbiology and Infectious Diseases, School of Pathology, University of the Witwatersrand, Faculty of Health Sciences, Johannesburg, South Africa | South Africa | Data collection | |
| Karen | Petersen | | MD | Department of Paediatrics, Chris Hani Baragwanath Academic Hospital, Faculty of Health Sciences, University of the Witwatersrand, Johannesburg, South Africa | South Africa | Data collection | |
| Martin | Hale | | MD | National Health Laboratory Service, Department of Anatomical Pathology, School of Pathology, University of the Witwatersrand, Faculty of Health Sciences, Johannesburg, South Africa | South Africa | Data collection | |
| Nelesh | P. Govender | | MD | National Institute for Communicable Diseases (NICD), Johannesburg, South Africa | South Africa | Data collection | |
| Peter | J. Swart | | MD | National Health for Laboratory Service in South Africa | South Africa | Data collection | |

**Supplement 2.** Nonauthor Collaborators
*First name, last name, and suffix (if applicable) are required and will appear in PubMed.

| ***First Name and Middle Initial(s)** | ***Last Name** | ***Suffix (eg, Jr, III)** | Academic Degrees | Institution | Location (city, state/province, country) | Role or Contribution, eg, chair, principal investigator | Group (if more than 1 Group listed in the byline) and/or Subgroup (eg, Steering Committee) |
|---|---|---|---|---|---|---|---|
| Sanjay | G. Lala | | MD | Department of Paediatrics and Perinatal HIV Research Unit, Chris Hani Baragwanath Academic Hospital, Faculty of Health Sciences, University of the Witwatersrand, Johannesburg, South Africa | South Africa | Data collection | |
| Sithembiso | Velaphi | | PhD | Department of Pediatrics, Chris Hani Baragwanath Academic Hospital, School of Clinical Medicine, Faculty of Health Sciences, University of the Witwatersrand, Johannesburg, South Africa | South Africa | Data collection | |
| Richard | Chawana | | PhD | South African Medical Research Council Vaccines and Infectious Diseases Analytics Research Unit, University of the Witwatersrand, Johannesburg, South Africa | South Africa | Data collection | |
| Yasmin | Adam | | MD | 1) Department of Obstetrics & Gynaecology, Chris Hani Baragwanath Academic Hospital, School of Clinical Medicine, 2) Faculty of Health Sciences, Univeristy of the Witwatersrand, Faculty Health Sciences, Johannesburg, South Africa | South Africa | Data collection | |
| Amy | Wise | | MSc | South African Medical Research Council Vaccines and Infectious Diseases Analytics Research Unit, University of the Witwatersrand, Johannesburg, South Africa | South Africa | Data collection | |

**Supplement 2.** Nonauthor Collaborators
*First name, last name, and suffix (if applicable) are required and will appear in PubMed.

| ***First Name and Middle Initial(s)** | ***Last Name** | ***Suffix (eg, Jr, III)** | Academic Degrees | Institution | Location (city, state/province, country) | Role or Contribution, eg, chair, principal investigator | Group (if more than 1 Group listed in the byline) and/or Subgroup (eg, Steering Committee) |
|---|---|---|---|---|---|---|---|
| Nellie | Myburgh | | PhD | South African Medical Research Council Vaccines and Infectious Diseases Analytics Research Unit, University of the Witwatersrand, Johannesburg, South Africa | South Africa | Data collection | |
| Sanwarul | Bari | | MD | Maternal and Child Health Division, International Center for Diarrhoeal Diseases Research (icddr,b), Dhaka, Bangladesh | Bangladesh | Data collection | |
| Shahana | Parveen | | MSS | International Centre for Diarrhoeal Disease Research, Bangladesh (icddr,b) | Bangladesh | Data collection | |
| Mohammed | Kamal | | PhD | Bangabandhu Sheikh Mujib Medical University, Dhaka, Bangladesh | Bangladesh | Data collection | |
| A.S.M. Nawshad | Uddin Ahmed | | FCPS | Bangladesh Institute of Child Health at Dhaka University and Dhaka Shishu Children's Hospital | Bangladesh | Data collection | |
| Mahbubul | Hoque | | FCPS | Bangladesh Institute of Child Health at Dhaka University and Dhaka Shishu Children's Hospital | Bangladesh | Data collection | |
| Saria | Tasnim | | FCPS | Dhaka Community Medical College and Hospital | Bangladesh | Data collection | |
| Ferdousi | Islam | | FCPS | Popular Medical College and Hospital in Dhaka, Bangladesh | Bangladesh | Data collection | |
| Farida | Ariuman | | FCPS | National Institute of Cancer Research and Hospital (NICRH), Dhaka, Bangladesh | Bangladesh | Data collection | |
| Mohammad | Mosiur Rahman | | MD | Bangabandhu Sheikh Mujib Medical University, Dhaka, Bangladesh | Bangladesh | Data collection | |
| Ferdousi | Begum | | MD | Bangabandhu Sheikh Mujib Medical University (BSMMU) | Bangladesh | Data collection | |

**Supplement 2.** Nonauthor Collaborators

*First name, last name, and suffix (if applicable) are required and will appear in PubMed.

| ***First Name and Middle Initial(s)** | ***Last Name** | ***Suffix (eg, Jr, III)** | Academic Degrees | Institution | Location (city, state/province, country) | Role or Contribution, eg, chair, principal investigator | Group (if more than 1 Group listed in the byline) and/or Subgroup (eg, Steering Committee) |
|---|---|---|---|---|---|---|---|
| K. Zaman | Zaman | | PhD | International Centre for Diarrhoeal Disease Research, Bangladesh (icddr,b) | Bangladesh | Data collection | |
| Mustafizur | Rahman | | PhD | International Centre for Diarrhoeal Disease Research, Bangladesh (icddr,b) | Bangladesh | Data collection | |
| Dilruba | Ahmed | | PhD | International Centre for Diarrhoeal Disease Research, Bangladesh (icddr,b) | Bangladesh | Data collection | |
| Meerjady | Sabrina Flora | | PhD | Institute of Epidemiology, Disease Control, and Research (IEDCR), Dhaka, Bangladesh | Bangladesh | Data collection | |
| Tahmina | Shirin | | PhD | Institute of Epidemiology, Disease Control and Research (IEDCR) | Bangladesh | Data collection | |
| Mahbubur | Rahman | | MPH | Institute of Epidemiology, Disease Control and Research (IEDCR) | Bangladesh | Data collection | |
| Joseph | Oundo | | PhD | 1) London School of Hygiene & Tropical Medicine, United Kingdom, 2) College of Health and Medical Sciences, Haramaya University, Harar, Ethiopia | Ethiopia | Data collection | |
| Alexander M. | Ibrahim | | MD | College of Health and Medical Sciences at Haramaya University | Ethiopia | Data collection | |
| Fikremelekot | Temesgen | | MD | Addis Ababa University | Ethiopia | Data collection | |
| Tadesse | Gure | | MD | College of Health and Medical Sciences at Haramaya University | Ethiopia | Data collection | |
| Addisu | Alemu | | MD | College of Health and Medical Sciences at Haramaya University | Ethiopia | Data collection | |
| Melisachew | Mulatu Yeshi | | MD | Ayder Specialized Comprehensive Hospital at Mekelle University | Ethiopia | Data collection | |
| Mahlet | Abayneh Gizaw | | MD | St. Paul's Hospital Millennium Medical College in Addis Ababa, Ethiopia | Ethiopia | Data collection | |
| Stian | MS Orlien | | PhD | London School of Hygiene & Tropical Medicine | Ethiopia | Data collection | |

**Supplement 2.** Nonauthor Collaborators
*First name, last name, and suffix (if applicable) are required and will appear in PubMed.

| ***First Name and Middle Initial(s)** | ***Last Name** | ***Suffix (eg, Jr, III)** | Academic Degrees | Institution | Location (city, state/province, country) | Role or Contribution, eg, chair, principal investigator | Group (if more than 1 Group listed in the byline) and/or Subgroup (eg, Steering Committee) |
|---|---|---|---|---|---|---|---|
| Solomon | Ali | | PhD | National Data Management Centre at the Ethiopian Public Health Institute | Ethiopia | Data collection | |
| Kitiezo | Aggrey Igunza | | BSc | Maseno University, Kenya | Kenya | Data collection | |
| Peter | Otieno | | MA | The University of Nairobi, Kenya | Kenya | Data collection | |
| Peter | Nyamthimba Onyango | | MA | The University of Nairobi, Kenya | Kenya | Data collection | |
| Janet | Agaya | | MPH | Maseno University, Kenya | Kenya | Data collection | |
| Richard | Oliech | | Diploma in | Kenya Polytechnic | Kenya | Data collection | |
| Joyce | Akinyi Were | | MSc | University of Nairobi, Kenya | Kenya | Data collection | |
| Dickson | Gethi | | BSc | University of Nairobi, Kenya | Kenya | Data collection | |
| George | Aol | | MA | Great Lakes University of Kisumu, Kenya | Kenya | Data collection | |
| Thomas | Misore | | MA | The University of Nairobi, Kenya | Kenya | Data collection | |
| Harun | Owuor | | MSc | Jaramogi Oginga Odinga University of Science and Technology, Kenya | Kenya | Data collection | |
| Christopher | Muga | | BSc | Jaramogi Oginga Odinga University of Science and Technology, Kenya | Kenya | Data collection | |
| Bernard | Oluoch | | Diploma in | Kenya Medical Training Institute, Nyeri, Kenya | Kenya | Data collection | |
| Christine | Ochola | | Diploma in | Kenya Medical Training Institute, Nyeri, Kenya | Kenya | Data collection | |
| Sharon | M. Tennant | | PhD | University of Maryland School of Medicine, Baltimore, Maryland, USA | Mali | Data collection | |
| Carol | L. Greene | | MD | University of Maryland School of Medicine, Baltimore, Maryland, USA | Mali | Data collection | |

**Supplement 2.** Nonauthor Collaborators
*First name, last name, and suffix (if applicable) are required and will appear in PubMed.

| ***First Name and Middle Initial(s)** | ***Last Name** | ***Suffix (eg, Jr, III)** | Academic Degrees | Institution | Location (city, state/province, country) | Role or Contribution, eg, chair, principal investigator | Group (if more than 1 Group listed in the byline) and/or Subgroup (eg, Steering Committee) |
|---|---|---|---|---|---|---|---|
| Ashka | Mehta | | MPH | Department of Pediatrics, Center for Vaccine Development and Global Health, University of Maryland School of Medicine, Baltimore, Maryland, USA | Mali | Data collection | |
| J. Kristie | Johnson | | PhD | University of Maryland School of Medicine, Baltimore, Maryland, USA | Mali | Data collection | |
| Brigitte | Gaume | | PhD | Center for Vaccine Development and Global Health, University of Maryland School of Medicine, Baltimore, Maryland, USA | Mali | Data collection | |
| Adama | Mamby Keita | | MD | Centre pour le Développement des Vaccins (CVD-Mali), Ministère de la Santé | Mali | Data collection | |
| Rima | Koka | | MD | University of Maryland School of Medicine, Baltimore, Maryland, USA | Mali | Data collection | |
| Karen | D. Fairchild | | MD | University of Virginia | Mali | Data collection | |
| Diakaridia | Kone | | MD | CSRef Commune I, Bamako, Mali | Mali | Data collection | |
| Diakaridia | Sidibe | | MD | Centre pour le Développement des Vaccins (CVD-Mali), Ministère de la Santé | Mali | Data collection | |
| Doh | Sanogo | | MD | Epidemiology Department CVD-Mali, Bamako, Mali | Mali | Data collection | |
| Uma U. | Onwuchekwa | | MSc | Bioinformatics department, CVD-Mali, Bamako, Mali | Mali | Data collection | |
| Nana | Kourouma | | MD, PHD | CVD-Mali, HGT, Bamako, Mali | Mali | Data collection | |

**Supplement 2.** Nonauthor Collaborators
*First name, last name, and suffix (if applicable) are required and will appear in PubMed.

| ***First Name and Middle Initial(s)** | ***Last Name** | ***Suffix (eg, Jr, III)** | Academic Degrees | Institution | Location (city, state/province, country) | Role or Contribution, eg, chair, principal investigator | Group (if more than 1 Group listed in the byline) and/or Subgroup (eg, Steering Committee) |
|---|---|---|---|---|---|---|---|
| Seydou | Sissoko | | MD | CVD-Mali, HGT, Bamako, Mali | Mali | Data collection | |
| Cheick | Bougadari Traore | | MD | CHU POINT G, Bamako Mali | Mali | Data collection | |
| Jane | Juma | | Ms, HND in | CVD-Mali, HGT, Bamako, Mali | Mali | Data collection | |
| Kounandji | Diarra | | MSc | CNAM/CVD-Mali, Bamako, Mali | Mali | Data collection | |
| Awa | Traore | | MSc | CNAM/CVD-Mali, Bamako, Mali | Mali | Data collection | |
| Tiéman | Diarra | | PhD, Profes | Point-Sud, Bamako, Mali | Mali | Data collection | |
| Kiranpreet | Chawla | | MD | Department of Obstetrics, Gynecology and Reproductive Sciences, University of Maryland School of Medicine, Baltimore, Maryland, USA | Mali | Data collection | |
| Tacilta | Nhampossa | | | | Mozambique | Data collection | |
| Zara | Manhique | | | | Mozambique | Data collection | |
| Sibone | Mocumbi | | | | Mozambique | Data collection | |
| Clara | Menéndez | | | | Mozambique | Data collection | |
| Khátia | Munguambe | | | Centro de Investigacao em Saude de Manhica<br>Eduardo Mondlane University, Faculty of Medicine, Community Health Department, Maputo, Mozambique | Mozambique | Data collection | |
| Ariel | Nhacolo | | | Centro de Investigação em Saúde de Manhiça [CISM] | Mozambique | Data collection | |
| Maria | Maixenchs | | | IS Global Hospital Clinic--Universitat de Barcelona, Spain<br>Centro de Investigacao en Saude de Manhica (CISM), Manhica, Mozambique | Mozambique | Data collection | |
| Andrew | Moseray | | MSc | Crown Agents | Sierra Leone | Data collection | |

**Supplement 2.** Nonauthor Collaborators
*First name, last name, and suffix (if applicable) are required and will appear in PubMed.

| ***First Name and Middle Initial(s)** | ***Last Name** | ***Suffix (eg, Jr, III)** | Academic Degrees | Institution | Location (city, state/province, country) | Role or Contribution, eg, chair, principal investigator | Group (if more than 1 Group listed in the byline) and/or Subgroup (eg, Steering Committee) |
|---|---|---|---|---|---|---|---|
| Fatmata | Bintu Tarawally | | MSc | FOCUS 1000 | Sierra Leone | Data collection | |
| Martin | Seppeh | | BSc | FOCUS 1000 | Sierra Leone | Data collection | |
| Ronald | Mash | | DrPH | Ministry of Health and Sanitation, Freetown, Sierra Leone | Sierra Leone | Data collection | |
| Julius | Ojulong | | MD | Crown Agents | Sierra Leone | Data collection | |
| Babatunde | Duduyemi | | FMCPath | University of Sierra Leone Teaching Hospital Complex, Freetown | Sierra Leone | Data collection | |
| James | Bunn | | MD | Human Development Team, British High Commission, Freetown | Sierra Leone | Data collection | |
| Alim | Swaray-Deen | | FWACS - O | University of Sierra Leone Teaching Hospital Complex, Freetown | Sierra Leone | Data collection | |
| Joseph | Bangura | | MPH | Ministry of Health and Sanitation, Freetown, Sierra Leone | Sierra Leone | Data collection | |
| Amara | Jambai | | MSc | Ministry of Health and Sanitation, Freetown, Sierra Leone | Sierra Leone | Data collection | |
| Margaret | Mannah | | MPH | Ministry of Health and Sanitation, Freetown, Sierra Leone | Sierra Leone | Data collection | |
| Okokon | Ita | | FMCPath - | University of Calabar Teaching Hospital, Nigeria | Sierra Leone | Data collection | |
| Cornell | Chukwuegbo | | FMCPath - | Federal Medical Center/PathConsult Diagnostics Ltd. Umuahia, Nigeria | Sierra Leone | Data collection | |

**Supplement 2.** Nonauthor Collaborators
*First name, last name, and suffix (if applicable) are required and will appear in PubMed.

| ***First Name and Middle Initial(s)** | ***Last Name** | ***Suffix (eg, Jr, III)** | Academic Degrees | Institution | Location (city, state/province, country) | Role or Contribution, eg, chair, principal investigator | Group (if more than 1 Group listed in the byline) and/or Subgroup (eg, Steering Committee) |
|---|---|---|---|---|---|---|---|
| Sulaiman | Sannoh | | MD | St. Luke's University Health Network, Easton, Pennsylvania, USA | Sierra Leone | Data collection | |
| Princewill | Nwajiobi | | FMCPath – | National Hospital, Abuja, Nigeria | Sierra Leone | Data collection | |
| Dickens | Kowuor | | MSc | Crown Agents | Sierra Leone | Data collection | |
| Erick | Kaluma | | MPH | Crown Agents | Sierra Leone | Data collection | |
| Oluseyi | Balogun | | MHM | Crown Agents | Sierra Leone | Data collection | |
| Carrie | Jo Cain | | RN | World Hope International, Makeni, Sierra Leone | Sierra Leone | Data collection | |
| Solomon | Samura | | BSc | World Hope International, Makeni, Sierra Leone | Sierra Leone | Data collection | |
| Samuel | Pratt | | MPH | FOCUS 1000 | Sierra Leone | Data collection | |
| Francis | Moses | | Master of M | Ministry of Health and Sanitation, Freetown, Sierra Leone | Sierra Leone | Data collection | |
| Tom | Sesay | | | Ministry of Health and Sanitation, Freetown, Sierra Leone | Sierra Leone | Data collection | |
| James | Squire | | MPhil Appl | Ministry of Health and Sanitation, Freetown, Sierra Leone | Sierra Leone | Data collection | |
| Joseph | Kamanda Sesay | | | Ministry of Health and Sanitation, Freetown, Sierra Leone | Sierra Leone | Data collection | |
| Osman | Kaykay | | MMed in C | Ministry of Health and Sanitation, Freetown, Sierra Leone | Sierra Leone | Data collection | |
| Binyam | Halu | | MPH | WHO | Sierra Leone | Data collection | |
| Hailemariam | Legesse | | Postgradua | UNICEF | Sierra Leone | Data collection | |

**Supplement 2.** Nonauthor Collaborators
*First name, last name, and suffix (if applicable) are required and will appear in PubMed.

| ***First Name and Middle Initial(s)** | ***Last Name** | ***Suffix (eg, Jr, III)** | Academic Degrees | Institution | Location (city, state/province, country) | Role or Contribution, eg, chair, principal investigator | Group (if more than 1 Group listed in the byline) and/or Subgroup (eg, Steering Committee) |
|---|---|---|---|---|---|---|---|
| Francis | Smart | | | Ministry of Health and Sanitation, Freetown, Sierra Leone | Sierra Leone | Data collection | |
| Sartie | Kenneh | | | Ministry of Health and Sanitation, Freetown, Sierra Leone | Sierra Leone | Data collection | |
| Soter | Ameh | | PhD | Crown Agents | Sierra Leone | Data collection | |
| Jana | Ritter | | DVM | Infectious Diseases Pathology Branch, Division of High-Consequence Pathogens and Pathology, National Center for emerging and Zoonotic Infectious Diseases, Centers for Disease Control and Prevention, Atlanta, US | PO / CPL | Data collection | |
| Tais | Wilson | | DVM | Centers for Disease Control and Prevention | PO / CPL | Data collection | |
| Jonas | Winchell | | PhD | Respiratory Diseases Branch, Division of Bacterial Diseases, National Center for Immunization and Respiratory Diseases, Centers for Disease Control and Prevention, Atlanta, US | PO / TAC | Data collection | |
| Jakob | Witherbee | | BS | Centers for Disease Control and Prevention | PO / TAC | Data collection | |
| Mischka | Garel | | MPH | Emory Global Health Institute, Emory University, Atlanta, Georgia | PO / 1599 | Data collection | |
| Navit | T. Salzberg | | MPH | Emory Global Health Institute, Emory University, Atlanta, Georgia | PO / 1599 | Data collection | |
| Jeffrey | P. Koplan | | MD | Emory Global Health Institute, Emory University, Atlanta, Georgia, USA | PO / 1599 | Data collection | |
| Kyu | Han Lee | | PhD | Emory Global Health Institute, Emory University, Atlanta, Georgia, USA | PO | Data collection | |

**Supplement 2.** Nonauthor Collaborators
*First name, last name, and suffix (if applicable) are required and will appear in PubMed.

| ***First Name and Middle Initial(s)** | ***Last Name** | ***Suffix (eg, Jr, III)** | Academic Degrees | Institution | Location (city, state/province, country) | Role or Contribution, eg, chair, principal investigator | Group (if more than 1 Group listed in the byline) and/or Subgroup (eg, Steering Committee) |
|---|---|---|---|---|---|---|---|
| Roosecelis | Martines | | | | CPL | Data collection | |
| Shamta | Warang | | | | CPL | Data collection | |
| Maureen | Diaz | | | | TAC | Data collection | |
| Jessica | Waller | | | | TAC | Data collection | |
| Shailesh | Nair | | MPH | Public Health Informatics Institute, The Task Force for Global Health, Atlanta, Georgia | PO | Data collection | |
| Lucy | Liu | | MBA | Public Health Informatics Institute at the Task Force for Global Health in Atlanta, Georgia, USA | PO | Data collection | |
| Courtney Bursuc | Liu | | MPH | Emory Global Health Institute, Emory University, Atlanta, GA, USA | PO | Data collection | |
| Kristin | LaHatte | | MA | Emory Global Health Institute, Emory University, Atlanta, GA, USA | PO | Data collection | |
| Sarah | Raymer | | BA | Emory Global Health Institute, Emory University, Atlanta, GA, USA | PO | Data collection | |
| John | Blevins | | ThD | Emory Global Health Institute, Emory University, Atlanta, GA, USA | PO | Data collection | |
| Solveig | Argeseanu | | PhD | Emory Global Health Institute, Emory University, Atlanta, GA, USA | PO | Data collection | |
| Kurt | Vyas | | PhD | Emory Global Health Institute, Emory University, Atlanta, GA, USA | PO | Data collection | |
| Manu | Bhandari | | MPH | Emory Global Health Institute, Emory University, Atlanta, GA, USA | PO | Data collection | |
