# Supplementary material for: Neurological Symptoms and Cause of Death Among Young Children in Low- and Middle-Income Countries
Source: JAMA Netw Open. 2024 Sep 3;7(9):e2431512. doi: 10.1001/jamanetworkopen.2024.31512 (PMC11372484; doi:10.1001/jamanetworkopen.2024.31512)
Supplement: Supplement 3. — Data Sharing Statement [file jamanetwopen-e2431512-s003.pdf]

Data Sharing Statement

Ajanovic. Neurological Symptoms and Cause of Death Among Young Children in Low- and Middle-Income Countries. *JAMA Netw Open*. Published September 03, 2024. doi:10.1001/jamanetworkopen.2024.31512

Data
Data available: Yes
Data types: Deidentified participant data
How to access data: https://champshealth.org
When available: With publication

Supporting Documents
Document types: None

Additional Information
Who can access the data: anyone requesting the data
Types of analyses: general data are available for any purpose
Mechanisms of data availability: general data are shared online in real time - open access. Data can be accessed at champshealth.org, and requests can be made at the site to access additional datasets. Datasets are publicly available at CHAMPS, 2021, “CHAMPS De-identified Dataset”; https://dataverse.unc.edu/dataset.xhtml?persistent Id=doi:10.15139/S3/PMAAWG).
